# Fine-Tuning Catalysts: The Role of Support Nanomorphology in Shaping Cu/CeO_2_ CO-PROX Properties

**DOI:** 10.1021/acscatal.5c06552

**Published:** 2025-11-25

**Authors:** Estefanía Fernández-Villanueva, Patricia Pérez-Bailac, Pablo G. Lustemberg, Ana B. Hungría, Laura Pascual, Renato Cataluña, Jose A. Vidal-Moya, Teresa Blasco, M. Verónica Ganduglia-Pirovano, Arturo Martínez-Arias

**Affiliations:** † 83076 Instituto de Catálisis y Petroleoquímica, CSIC, C/Marie Curie 2., 28049 Madrid, Spain; ‡ Universitat Politècnica de València, Camí de Vera s/n, 46022 Valencia, Spain; § Departamento de Ciencia de Materiales, Ingeniería Metalúrgica y Química Inorgánica, Facultad de Ciencias, Universidad de Cádiz, 11510 Cádiz, Spain; ∥ Departamento de Físico-Química, 28124 Universidade Federal do Rio Grande do Sul, Porto Alegre, Rio Grande do Sul BR-91501970, Brazil; ⊥ 83167 Instituto de Tecnología Química, Universitat Politècnica de València – Consejo Superior de Investigaciones Científicas (UPV-CSIC), 46022 Valencia, Spain

**Keywords:** Cu/CeO_2_ catalysts, hydrogen, CO-PROX, DFT, electron microscopy, carbonyls infrared

## Abstract

Understanding how oxide nanomorphology directs metal–support interactions is key to designing selective, low-cost catalysts. The preferential oxidation of CO (CO-PROX) is vital for purifying H_2_ streams for fuel cell applications, as even trace amounts of CO strongly poison the electrode catalysts. Cu/CeO_2_ systems provide a cost-effective alternative to noble metals, yet the influence of ceria morphology on the performance remains unclear. Here, we compare low-loaded Cu catalysts supported on CeO_2_ nanospheres and nanocubes. Although distinct in shape, electron microscopy, low-temperature CO adsorption infrared spectroscopy, and DFT calculations reveal surface reconstructions in nanocubes that diminish structural differences between the two supports. Nevertheless, the Cu/nanosphere catalyst shows higher CO oxidation activity, while the Cu/nanocube catalyst offers superior CO_2_ selectivity and a broader full-conversion temperature window. In situ DRIFTS and DFT spectra attribute these contrasts to stronger CO adsorption sites in the nanocube system. Copper speciation and the nature of surface carbonyls were resolved through complementary techniques, including STEM-HAADF imaging, XEDS mapping, EPR, and CO adsorption IR spectroscopy, together with DFT. These results demonstrate that subtle variations in ceria morphology steer interfacial chemistry and reaction pathways, providing design principles for next-generation Cu-based catalysts for CO-PROX and related oxidation reactions.

## Graphical abstract

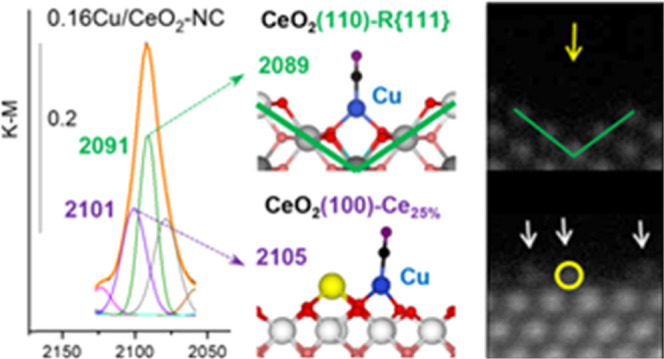

## Introduction

Catalysts combining copper and ceria (CeO_2_) have emerged as highly effective systems for a variety of C_1_ chemistry processes, owing to their unique redox and catalytic properties.^1–5^ Among these, the preferential oxidation of CO in H_2_-rich streams (CO-PROX) stands out as a particular relevant application, critical for purifying hydrogen produced via hydrocarbon reforming.^6^ Remarkably, Cu/CeO_2_ catalysts exhibit CO-PROX activity and selectivity comparable to noble metals while benefiting from their lower cost and greater availability.

The superior performance of these catalysts is largely attributed to the distinctive properties of the Cu–CeO_2_ interface, where strong metal–support interactions promote favorable redox behavior and catalytic activity in CO-PROX.^6–13^ The interfacial properties are strongly influenced by the surface structure of the ceria support, particularly, the nature of the exposed crystal facets. The (111) facet is the most thermodynamically stable,^14^ whereas the (110) or (100) surfaces are more reactive but prone to reconstruction, depending on the nanocrystal morphology thermal history.^14–20^

Indeed, CeO_2_ nanostructures, such as cubes, rods, and spheres, expose distinct surface facets and exhibit markedly different catalytic behavior in CO oxidation, which has been correlated with their surface structure.^21–23^ In addition to the support morphology, catalytic performance also depends on the nature of the dispersed copper species, which can range from single isolated atoms to CuO_*x*_ clusters or CuO_*x*_ particles.^24,25^

Despite significant advances in the synthesis of shape-controlled CeO_2_ and the characterization of Cu species, the origin of the structure–performance relationship in Cu/CeO_2_ CO-PROX catalysts remains poorly understood. In particular, it is unclear how different nanomorphologies of ceria, after undergoing possible surface reconstruction, affect the reactivity and selectivity of supported copper species under the CO-PROX conditions. A clearer understanding of these effects is essential to rational catalyst design, especially under low-Cu-loading regimes where interfacial interactions dominate.

In this study, we investigate copper catalysts supported on CeO_2_ with two distinct nanomorphologies: nanospheres (Cu/CeO_2_–NS) and nanocubes (Cu/CeO_2_–NC), with low copper loading to maximize the interaction between the copper species and the support. Following significant reconstruction observed in the nanocubes, both supports exhibit important similarities in the types of exposed ceria surfaces. However, notable catalytic and chemical differences are observed between the supported copper catalysts. Cu/CeO_2_–NS demonstrates the highest CO oxidation activity but also the lowest selectivity toward CO_2_. In contrast, the nanocube-supported samples exhibit lower CO oxidation activity but are significantly more selective to CO_2_ compared to Cu/CeO_2_–NS. As a consequence, a wide full CO conversion temperature window is exclusively observed for Cu/CeO_2_–NC. The differences can be attributed to the strong CO adsorption sites that are unique to the Cu/CeO_2_–NC samples, which enhance the CO_2_ selectivity and influence the overall reaction pathway.

These findings provide new insight into the role of support nanomorphology in tuning interfacial reactivity and selectivity and contribute to the development of structure–activity guidelines for the rational design of Cu-based catalysts for CO-PROX and related oxidation reactions.

## Experimental and Theoretical Details

CeO_2_ nanospheres and nanocubes were synthesized using microemulsion and hydrothermal methods, respectively, as previously described.^21,22^ Copper (1 wt %) was deposited on both supports by incipient wetness impregnation using copper nitrate aqueous solutions, resulting in catalysts denoted as 1Cu/CeO_2_–NS and 1Cu/CeO_2_–NC. Additionally, a third catalyst, 0.16Cu/CeO_2_–NC, containing 0.16 wt % copper, was prepared using the same method. In any case, the catalysts were finally calcined under air at 500 °C for 2 h; full details can be found elsewhere.^21,22^ The composition of 0.16Cu/CeO_2_–NC was chosen to match the theoretical surface density of copper to that of 1Cu/CeO_2_–NS, accounting for differences in the specific surface area of the supports. The copper loading employed is relatively low in order to maximize the interactions with the CeO_2_ supports. Taking into account the *S*_BET_ of the supports (reported in the Supporting Information), it corresponds theoretically to about one and six coppers per every nine ceriums exposed at the surface for 1Cu/CeO_2_–NS (or 0.16Cu/CeO_2_–NC) and 1Cu/CeO_2_–NC, respectively.

High-resolution transmission electron microscopy (HRTEM) data were recorded on a JEOLTEM/STEM2100F field emission gun transmission electron microscope operating at 200 kV. Scanning transmission electron microscopy with high-angle annular dark field (STEM-HAADF) imaging and high spatial resolution energy-dispersive X-ray spectroscopy (XEDS) mapping were conducted using a double aberration corrected FEI Titan3 Themis 60–300 microscope, equipped with a four-detector ChemiSTEM system. A high brightness, subangstrom diameter electron probe, combined with a highly stable stage, was used to acquire the images and maps. Elemental mapping was performed with a screen current of 200 pA and a pixel dwell time of 170 μs, resulting in a frame acquisition time of approximately 25 s. Drift correction was applied using cross-correlation, and image processing was carried out using an averaging filter available in the Esprit software. Electron microscopy samples were prepared by depositing small amounts of catalyst powder onto holey carbon-coated gold grids.

In situ diffuse reflectance infrared Fourier transform spectroscopy (DRIFTS) experiments were conducted by using a Bruker Equinox 55 FTIR spectrometer equipped with an MCT detector. A Harrick low-temperature cell cooled with liquid N_2_ was employed. Approximately 100 mg of each sample was pretreated in 10% O_2_/N_2_ at 673 K, followed by cooling under pure N_2_ to the lowest possible temperature, at which point 1% CO was introduced in the N_2_ flow. Flows of 100 mL min^–1^ were employed in any case. The carbonyl species observed during the experiments were analyzed by least-squares fitting using pure Gaussian line shapes, as expected for the adsorbed species detected.

EPR spectra were recorded with a Bruker EMX-12 spectrometer operating at the X-band, using a modulation frequency of 100 kHz and an amplitude modulation of 1G. All spectra were measured at 100 K. Quantitative analysis was carried out by double integration of the spectra using CuSO_4_ as an external reference standard.

Catalytic activity measurements under CO-PROX conditions were conducted in a tubular reactor using a gas mixture of 1% CO, 1.25% O_2_, and 50% H_2_ (including 0.1% H_2_O) in He, at a total flow rate of 100 mL min^–1^. Typically, 100 mg of the catalyst was physically mixed with 1 g of inert SiC (Merck) to fill the reactor. All samples were pretreated in 10% of O_2_ in He before testing. An additional experiment was conducted by varying the sample weight to evaluate the performance at an equivalent copper content. Reaction products were detected using a gas-phase infrared cell (from Infrared Analysis Inc.) combined with online mass spectrometry (Pfeiffer Omnistar).

Density functional calculations (DFT) were performed using the slab-supercell approach, implemented in the Vienna Ab-initio Simulation Package (VASP, http://www.vasp.at; VASP version 5.4.4).^26–29^ The Ce (4f, 5s, 5d, 6s), O (2s, 2p), Cu (3p, 3d, 4s), and C (2s, 2p) electrons were treated explicitly as valence states within the projector-augmented-wave (PAW) framework, with a plane-wave energy cutoff of 500 eV. The remaining electrons were included in the atomic cores.

Structural optimizations and vibrational frequency calculations were carried out using the hybrid HSE06 functional.^30^ To reduce computational cost, Brillouin zone integration was restricted to the Γ *k*-point, and structures were initially preoptimized using the PBE + U method, employing the same Γ-point sampling and the cell parameters obtained from HSE06 optimizations. The PBE + U approach, used for structural preoptimization, combines the Perdew–Burke–Ernzerhof (PBE) generalized gradient approximation (GGA) functional with an on-site Hubbard U correction.^31,32^ A U value of 4.5 eV was applied specifically to the Ce 4f states. Convergence criteria were set to 10^–6^ eV for the self-consistent field energy and 0.01 eV/Å for the maximum force acting on any atom.

The CeO_2_(111), (100), and (110) surfaces were modeled using (3 × 3), c(2 × 2), and (2 × 4) periodicities, respectively, and consisted of six, nine, and nine atomic layers. These slabs were derived from bulk ceria using a calculated equilibrium lattice parameter of *a*_0_ = 5.398 Å, as obtained at the HSE06 level. For the polar bulk-truncated (100), an O-terminated *c*(2 × 2) configuration, commonly referred to as the checkerboard model and labeled (100)-O, was employed to compensate for polarization. In this model, only half of the oxygen atoms occupy the topmost layer, while the remaining half are positioned at the bottom of the slab (Figure S1).

Additionally, reconstructed (100) surfaces with pyramid-like features, labeled (100)-Ce, were generated by manually adding CeO_2_ units to the checkerboard (100)-O model. This modification results in Ce atoms at the tops of the pyramids being 4-fold coordinated (Figure S1). Mixed terminations, labeled (100)-Ce_25%_ and (100)-Ce_50%_, were constructed by adding one or two CeO_2_ units, respectively. These partially reconstructed surfaces have been reported to be more stable than the pure (100)-O termination and were therefore also included in this study.^33,34^

For the nonpolar (110) surface, a sawtooth-like (110) reconstruction has been observed.^17,19^ The corresponding model, labeled (110)-R{111}, was constructed by cutting the (110) slab along (111) planes to reproduce this structural motif (Figure S1).

Finally, to mimic the interaction between two adjacent nanocubes exposing (100)-O terraces, a stepped surface model was created. This was achieved by cutting a larger (110) slab along (100) planes, thereby generating a step edge composed of two nearly perpendicular (100)-O facets (Figure S1).

Surface models with Cu atoms or Cu_4_ clusters were constructed by placing the adsorbates on specific surface sites. During geometry optimizations, the bottommost Ce and O atomic layers of each slab were fixed to simulate bulk constraints. Isolated molecules were optimized in a 15 × 15 × 15 Å^3^ cubic box by using only the Γ *k*-point for Brillouin zone sampling.

The average adsorption energy of CO at two different coverages was calculated using the following expression:

1 $${\Delta E}_{ads}=1/n\left(E_{CO/Surface}-E_{Surface}-n\cdot E_{Molecule\left(gas\right)}\right)$$

where *E*_CO/Surface_ is the total energy of the surface with *n* adsorbed CO molecules, *E*_Surface_ is the energy of the clean surface, and *E*_Molecule(gas)_ is the energy of a single CO molecule in the gas phase. The binding energy of Cu to the ceria surfaces was calculated analogously.

Vibrational frequencies and normal modes were obtained numerically using central finite differences of analytically calculated forces, as implemented in VASP. Atomic displacements of ±0.015 Å were applied in partial Hessian calculations with all slab atoms kept fixed. Reported vibrational frequencies were scaled by a factor λ = ν_exp_/ν_calc_, where ν_exp_ = 2143 cm^–1^ is the experimental gas-phase frequency of CO and λ_HSE06_ = 2234 cm^–1^ is the corresponding value computed with the HSE06 functional. Infrared (IR) intensities for each normal mode were computed as the square of the first derivative of the z-component of the dynamic dipole moment. Atomic charges were determined using Bader charge analysis.^35–37^ The Jmol and p4vasp software packages were used to construct and visualize atomic structures and vibrational modes,^38,39^ while VESTA was employed to generate high-quality images of the atomic models.^40^

## Results and Discussion

### Support Characterization

The characterization of the supports, including specific surface area (*S*_BET_), X-ray diffraction (XRD), and Raman spectroscopy, was reported in a previous study and is summarized in Table S1 of the Supporting Information.^21^ Transmission electron microscopy (TEM) images of the two supports are presented in Figure 1, revealing distinct morphologies resulting from differences in synthesis parameters, as described in the Experimental and Theoretical Details section. These morphologies include nanocubes (CeO_2_–NC) and nanospheres or nanopolyhedra (CeO_2_–NS). In principle, the CeO_2_–NC structures are expected to predominantly expose (100) crystal facets,^19^ whereas CeO_2_–NS likely exposes a mixture of surface terminations, with the thermodynamically stable (111) facet being the most abundant.^41^ HRTEM images in Figure 1 are consistent with the nanospheres exposing (111) facets that appear flat at the atomic scale, as seen in the magnified views and their corresponding FFTs along the [101] zone axis. For the nanocubes, the HRTEM image shows a particle oriented along the [101] zone axis too, as confirmed by its FFT. The band-pass filtered image (bottom right) enhances surface detail, apparently revealing a pronounced sawtooth-like arrangement of short (111) steps along the (110) edge, indicative of significant surface faceting and reconstruction,^19^ as will be further explored below.

**Figure 1:**
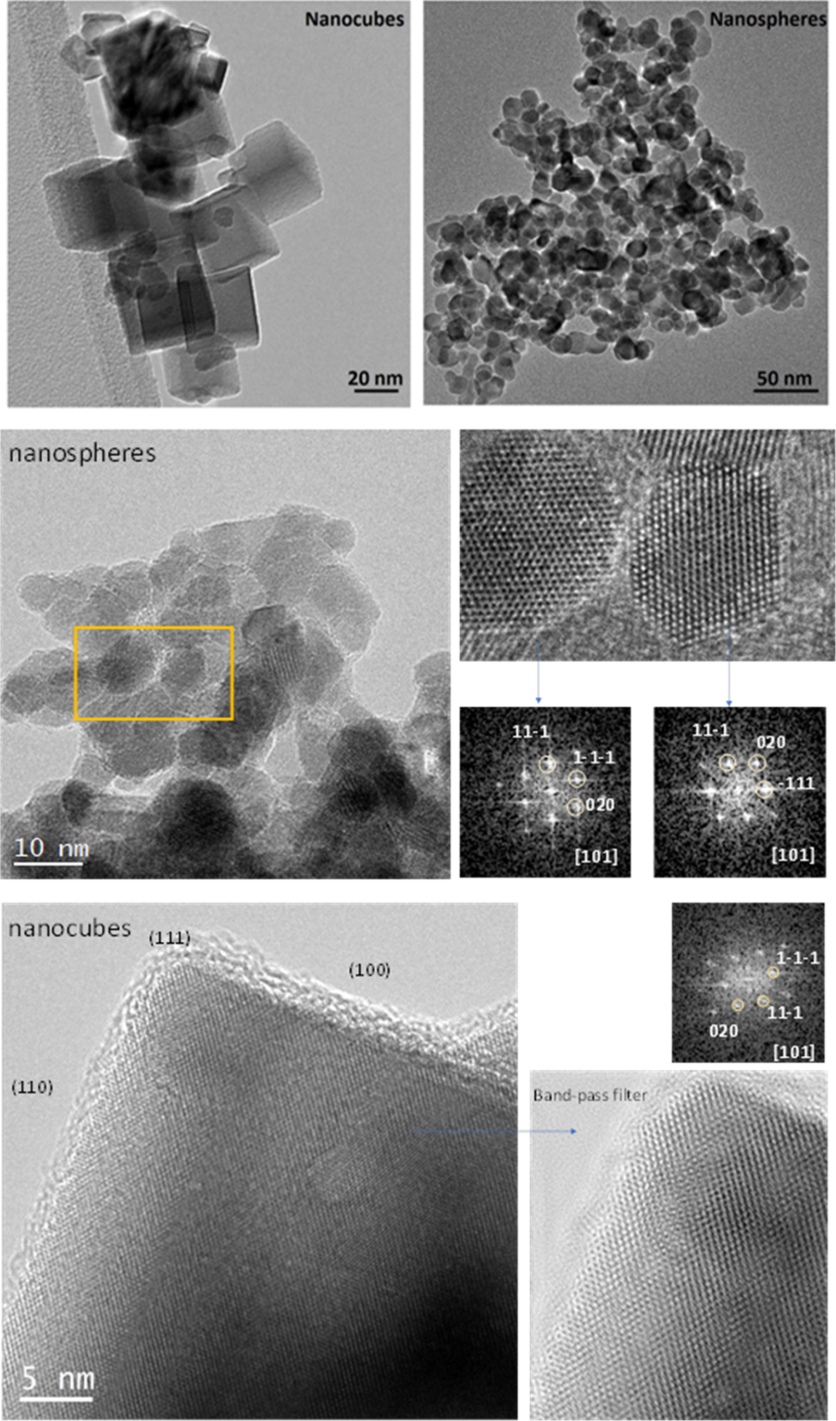
TEM pictures of the two CeO_2_ supports (top). HRTEM images of CeO_2_ nanospheres (middle) and nanocubes (bottom). Diffraction diagrams taken on indicated particles are shown.

To gain deeper insight into the nature of the exposed surface facets, we draw inspiration from recent studies by Yang et al. and Lustemberg et al. as well as a recent study by Caulfield et al.^17,19,42–44^ These studies provided systematic and detailed characterizations of CeO_2_ single-crystal surfaces using infrared reflection–absorption spectroscopy (IRRAS) with low-temperature CO adsorption, complemented by DFT calculations employing the hybrid HSE06 functional. In this work, we applied peak fitting to the experimental DRIFTS spectra of the two CeO_2_ supports. The resulting component peaks show good agreement with the vibrational frequencies previously determined experimentally and assigned via DFT for CO adsorbed on oxidized, low-index CeO_2_ single-crystal surfaces,^17,19,42,43^ as shown in Figure 2 and summarized in Table 1.

**Figure 2:**
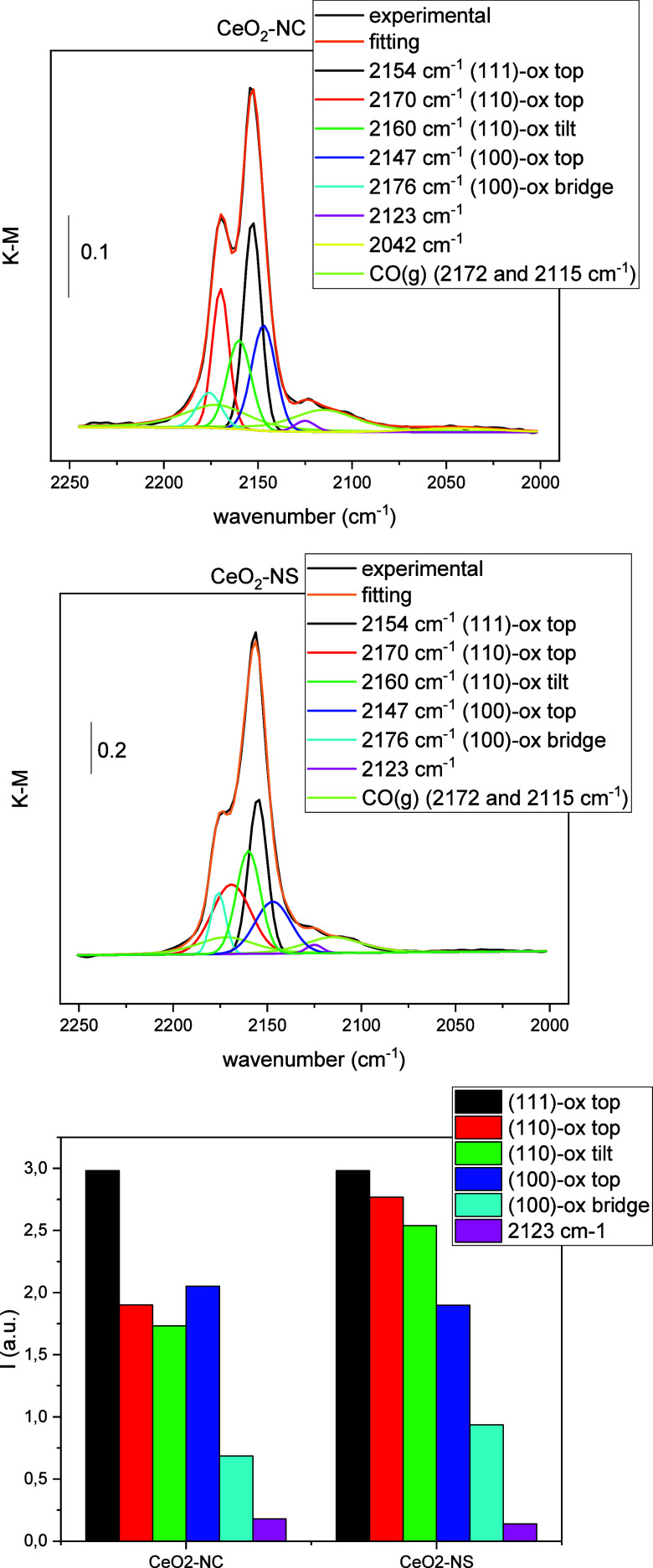
DRIFTS spectra recorded under 1% CO/N_2_ flow at 137 K over CeO_2_-NC (top) and CeO_2_-NS (middle) supports, pretreated under 10% O_2_/N_2_ at 653 K and subsequently cooled under N_2_ flow. The bottom plot shows the relative contributions of the indicated carbonyl species, with the intensity of the carbonyl over the (111) face used as the normalization reference.

**Table 1:** CO infrared IRRAS data and corresponding adsorption mode assignments on oxidized low-index CeO_2_ single-crystal surfaces, as reported in Ref 43

surface	IRRAS	Ads. Mode
CeO_2_(111)	2154	Top
CeO_2_(100)-O	2176	Bridge
	2147	Top
CeO_2_(110)	2170	Top
	2160	Tilt

Despite the differences in the structures of the nanoforms (Figure 1), notable similarities are observed between the two supports in the spectra of CO adsorption (Figure 2). In both CO adsorption spectra, a prominent band appears near 2154 cm^–1^, followed in intensity by three other significant features at approximately 2170, 2160, and 2147 cm^–1^. These bands correspond to CO adsorption on oxidized ceria surfaces, specifically: top-site adsorption on CeO_2_(111), top-site and tilted adsorption on CeO_2_(110), and bridge-site adsorption on CeO_2_(100)-O (Table 1). Additionally, weaker bands are detected at lower frequencies (2123 and 2042 cm^–1^), which are likely attributable to carbonyl species adsorbed at defect sites such as corners, edges, or oxygen vacancies in the nanocrystalline powders. These features are notably absent in the spectra of larger single-crystal models,^19^ further supporting their assignment to undercoordinated or defective surface sites. Nevertheless, we cannot discard the possibility that the band at 2123 cm^–1^ could in fact correspond to a Ce^3+^ electronic transition rather than a carbonyl, as proposed in a recent work.^45^

These results are consistent with expectations for nanospheres, as polyhedral morphologies typically expose multiple surface facets, with the thermodynamically stable (111) facet generally being the most dominant.^41^ However, in the case of nanocubes, carbonyl species associated with the (111) surface contribute significantly more than anticipated. This could be due to the fact that, as indicated by HRTEM (Figure 1), the (110) edges, as well as probably the (100) facets too, of the nanocubes may have undergone substantial faceting or reconstruction, resulting in the formation of nanostructured (111) (or (111)-like) surfaces.

It is important to note that the intensity of each carbonyl band depends not only on the relative amount of CO adsorbed on each surface facet under the given experimental conditions but also on the extinction coefficients of the corresponding vibrational modes. To the best of our knowledge, such coefficients are not currently available in the literature. Additional DRIFTS experiments conducted at varying CO adsorption temperature (Supporting Information Figure S2) further highlight the challenge of directly correlating band intensities with the abundance of specific surface facets.

Nevertheless, the occurrence of nanocube reconstruction is confirmed by high-resolution scanning transmission electron microscopy (STEM), which reveals a sawtooth-like arrangement of short, nearly perpendicular (111) facets aligned along the (110) terraces (Figure 3, indicated by yellow arrows on the left). This structural motif is in agreement with previous literature reports.^16,19,46^

**Figure 3:**
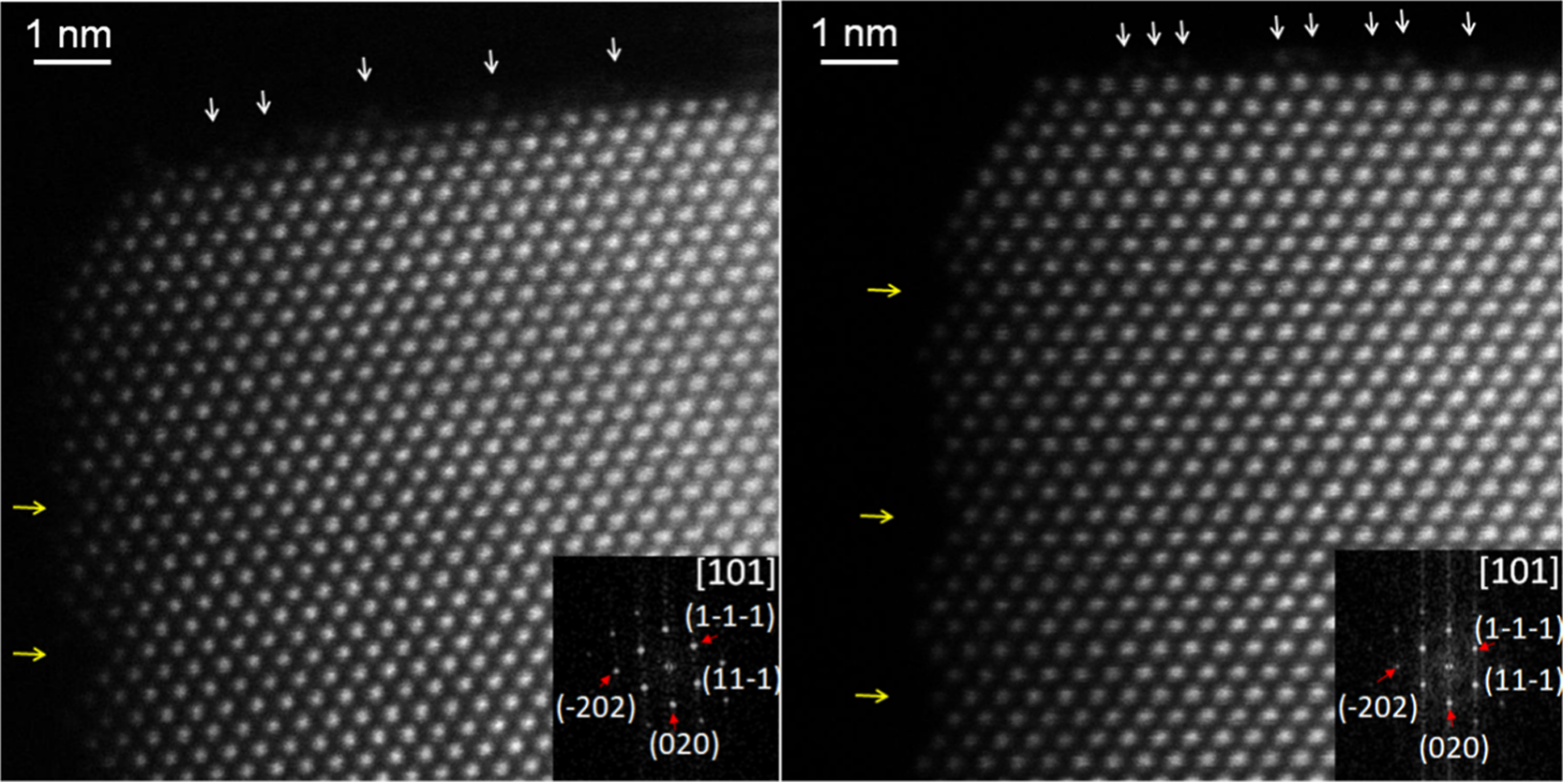
High-resolution STEM-HAADF images of two distinct nanocubes, showcasing the reconstruction of the (110) edges of the cube on {111}-type nanofacets (yellow arrows) and surface cerium atoms on top of the (100) face (white arrows).

Regarding the (100) facets, contrast patterns attributed to surface cerium atoms are also observed (Figure 3, marked with white arrows at the top). Such surface rearrangements of cerium atoms on (100) planes have been previously reported^15^ and are associated with atomic-scale mobility that can lead to relatively ordered reconstructions—as seen in the left nanocube—or more disordered configurations, as observed in the right nanocube. Notably, these reconstructions on (100) surfaces have also been characterized under nonbeam conditions using methanol adsorption infrared spectroscopy,^15^ confirming their intrinsic nature.

Based on the experimental evidence of surface reconstruction on the (110) and (100) facets of the nanocubes, three computational models—denoted as(110)-R{111}, (100)-Ce_25%_, and (100)-Ce_50%_—were developed to investigate CO adsorption (see the Theoretical Details section and Figure S1 in the Supporting Information). These models aim to complement both the previous computational study^43^ and the DRIFTS characterization presented in Figure 2. The results for a full monolayer CO coverage (i.e., one molecule per exposed adsorption site) are shown in Figure 4 and support the existence of reconstructed (110) and (100) surfaces on the nanocubes, as discussed below.

**Figure 4:**
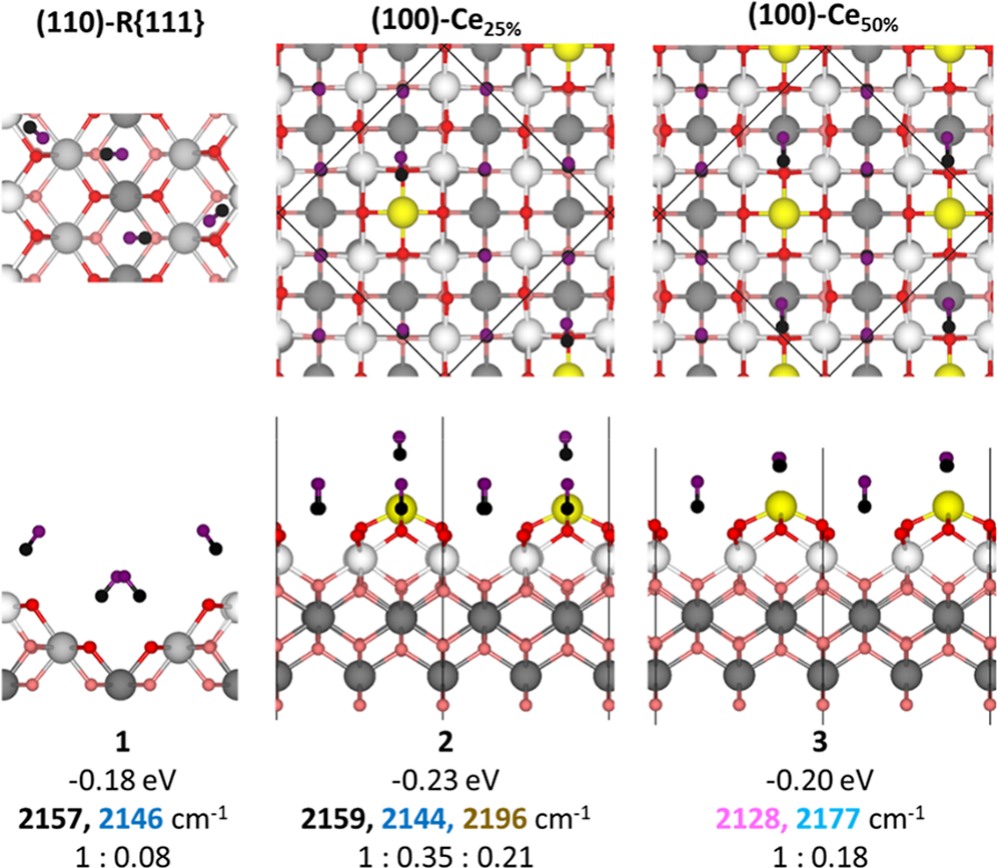
Top and side views of the optimized structures for the (110) and (100) reconstructions at full CO coverage (one molecule per exposed adsorption site in the surface unit cell). The average adsorption energies and corresponding CO vibrational frequencies, along with their relative intensities, are provided. Surface Ce and O atoms are shown in white and red, respectively, while deeper Ce and O atoms are depicted in gray and pink; O atoms from CO molecules are represented in purple, C atoms in black, and added Ce atoms forming pyramidal structures are highlighted in yellow. In cases where vibrational frequencies match the fitted component peaks in Figure 2, the corresponding colors are used to indicate this correlation.

On the (110)-R{111} surface—which exhibits a characteristic sawtooth zigzag geometry—three distinct CO adsorption modes are observed at low coverage (Figure S3). The most stable configuration (structure S1) corresponds to a CO molecule adsorbed in a tilted orientation at the crest of the sawtooth, closely resembling the most favorable adsorption site previously reported for the pristine (110) surface.^43^ The second configuration (structure S2) involves the addition of CO adsorbed in a vertical (top-down) orientation at the same crest site, corresponding to the second most stable mode on the (110) surface. In the third configuration (structure S3), the CO molecule is located in the valley of the sawtooth, adopting a tilted orientation. This geometry closely resembles that of CO adsorbed on a (111) surface; however, it should be noted that both the molecule and the local surface structure are tilted due to the faceted nature of the model.

At full CO coverage—comprising four CO molecules, thereby closely replicating the conditions used in the DRIFTS experiments—the first and third adsorption modes identified at low coverage (structures S1 and S3 in Figure S3) coexist in a single configuration (structure 1, Figure 4). This structure features two CO molecules adsorbed in tilted orientations at (110)-like sites and two adsorbed vertically at the top (111)-like sites.

The vibrational spectrum of this configuration exhibits a dominant band at 2157 cm^–1^ and a secondary, less intense feature at 2146 cm^–1^. While all CO molecules contribute to the overall vibrational profile, the 2157 cm^–1^ band arises primarily from CO in the tilted (110)-like adsorption mode, whereas the 2146 cm^–1^ band is mainly associated with CO adsorbed at the top (111)-like sites. These findings are consistent with the vibrational frequencies previously calculated for each mode at low CO coverage (Figure S3). Importantly, the most intense vibrational mode on the CeO_2_(110)-R­{111} surface (2157 cm^–1^) matches the frequency calculated for CO adsorbed on the CeO_2_(111) surface.^43^ However, in this case, the mode arises predominantly from CO adsorbed at tilted (110)-like sites rather than from the standard flat (111) surface.

Furthermore, the vibrational frequencies computed for the CeO_2_(110)-R­{111} surface are similar to those traditionally assigned to CO on unreconstructed (111), (110), and O-terminated (100) ceria surfaces,^17,19,43^ as summarized in Table 2. This overlap complicates unambiguous peak assignments in the experimental DRIFTS spectra. Nevertheless, the similarity is significant, as it suggests that frequency assignments based solely on the characteristics of extended, unreconstructed CeO_2_ surfaces may overlook the presence of reconstructed surface features—particularly in nanostructured materials.

**Table 2:** Summary of CO IRRAS Experimental Data, HSE06-Calculated Vibrational Frequencies, and Corresponding Adsorption Mode Assignments on Low-Index Oxidized CeO_2_ Single-Crystal Surfaces, as Reported in ref 43, along with HSE06 Results, Mode Assignments, and Relative Intensities for Reconstructed Oxidized CeO_2_ Surfaces from This Work

surface	IRRAS (cm^–1^)	HSE06 (cm^–1^)	adsorption mode	Rel. intensity	reference
CeO_2_(111)	2154	2157	top		^43^
CeO_2_(100)-O	2176	2176	bridge		^43^
	2147	2151	top		
CeO_2_(100)-Ce_25%_		2159	bridge (sym)	1.00	this work
		2144	bridge (asym)	0.35	
		2196	tilt (pyr)	0.21	
CeO_2_(100)-Ce_50%_		2128	bridge (sym)	1.00	this work
		2177	tilt (pyr)	0.18	
CeO_2_(110)	2170	2165	top		^43^
	2160	2150	tilt		
CeO_2_(110)-R{111}		2157	tilt (110)-like	1.00	this work
		2146	top (111)-like	0.08	

This comparison provides further insight into why experimental evidence of sawtooth-type surfaces has only emerged relatively recently.^16–19,46^ For example, Yang et al. reported that (110) planes characteristic of CeO_2_ nanorods undergo refaceting into {111} planes at elevated temperatures.^17^ This transformation resulted in the appearance of two distinct bands at 2154 and 2163 cm^–1^ in s-polarized IR spectra—features consistent with on-top CO adsorption on partially reduced CeO_2_(111) surfaces. The presence of these bands on surfaces originally identified as (110) strongly suggests that carbonyl species are adsorbed on {111} planes that are tilted with respect to the (110) orientation. This tilt allows for the activation of vibrational modes in s-polarized IR light, thereby enabling their detection. Such findings demonstrate that vibrational signatures traditionally attributed to flat, unreconstructed low-index surfaces may, in fact, arise from structurally reconstructed or faceted domains—particularly in nanostructured or thermally treated materials.

For the (100)-Ce surfaces, which feature CeO_4_ pyramid-like terminations with a Ce-terminated surface, two primary CO adsorption sites are identified—regardless of whether one or two pyramids are present (Figure 4). The first site corresponds to the exposed cerium atom at the apex of the pyramid (highlighted in yellow in structures **2** and **3**), while the second is a bridge site between two Ce atoms, previously identified as the most stable adsorption site on the checkerboard (100)-O surface.^33^

The concentration of pyramids significantly influences the distribution of adsorption sites and the resulting vibrational frequencies, as illustrated in structures **2** and **3** (Figure 4). Each added pyramid replaces two bridge sites with a single Ce-terminated site, reducing the total number of available adsorption sites and altering the pyramid-to-bridge site ratio in the (100)-Ce25% and (100)-Ce50% models. On the ideal (100)-O surface, eight bridge sites are available for CO adsorption. In the (100)-Ce_25%_ model, the introduction of one pyramid reduces this number to six, resulting in a maximum of seven adsorbed CO molecules and a 1:6 pyramid-to-bridge ratio. In contrast, the (100)-Ce_50%_ model includes two pyramids and accommodates six CO molecules in a 2:4 ratio.

The average CO adsorption energy decreases on (100)-Ce_50%_ compared to (100)-Ce_25%_, indicating a weaker overall interaction with the surface at higher pyramid concentration. Furthermore, the observed increase in C–O bond lengths (bridge: 1.131 vs 1.134 Å; pyramid: 1.125 vs 1.127 Å) correlates with a redshift in vibrational frequencies (bridge: 2159 vs 2128 cm^–1^; pyramid: 2196 vs 2177 cm^–1^ (Figure 4 and Table 2), consistent with bond softening due to electron backdonation and reduced C–O bond strength. In the (100)-Ce_50%_ model, CO molecules adsorbed at the Ce atoms of the pyramid sites also experience less steric hindrance due to the absence of neighboring bridge sites, allowing the molecules to tilt more visibly toward the surface (Figure 4).

Notably, the most intense vibrational mode for the (100)-Ce25% model arises from the CO molecules bound at bridge sites and appears at 2159 cm^–1^, closely matching the experimentally observed band at 2154 cm^–1^. Additionally, a weaker band predicted at 2144 cm^–1^—corresponding to asymmetric stretching of bridge CO molecules (structure 2, Figure 4 and Table 2)—agrees well with the experimental feature at 2147 cm^–1^.

These results add significant complexity to the interpretation of Figure 2. As previously noted, inferring the quantity of surface species from DRIFTS band intensities is challenging due to the unknown extinction coefficients for each vibrational mode. This complexity is further amplified when considering the potential presence of reconstructed surfaces, which introduce variations in local geometry and intensity of the associated vibrational modes. Moreover, the calculated vibrational frequencies for reconstructed CeO_2_(110)-R­{111} and (100)-Ce surfaces overlap with those of unreconstructed (111), (110), and checkerboard (100)-O ceria surfaces, complicating unambiguous peak assignments. Nevertheless, by combining the relative DRIFTS intensities in Figure 2 with theoretical predictions and structural insight, meaningful conclusions can still be drawn.

For ideal nanoshapes, lower (111)/(100) peak ratios are expected for nanocubes compared to those for nanospheres. The vibrational bands at 2154 and 2147 cm^–1^ are characteristic of on-top CO adsorption on the (111) and (100)-O surfaces, respectively.^18,43^ In our nanocube sample, however, the relative intensity of the 2154 cm^–1^ peak is significantly higher than that of the 2147 cm^–1^ peak—greater than anticipated, even when accounting for differences in specific surface area (Table S1, Supporting Information).

While the 2154 cm^–1^ band is conventionally attributed to CO on (111) facets, our computational results suggest that similar vibrational modes can also arise from reconstructed surfaces. Specifically, the most intense modes for the CeO_2_(110)-R­{111} and (100)-Ce25% models occur at 2157 and 2159 cm^–1^, respectively, with secondary bands at 2146 and 2144 cm^–1^ (Figure 4 and Table 2). These values closely match the dominant and shoulder peaks in the DRIFTS spectra (Figure 2), indicating that reconstructed facets may significantly contribute to the observed intensities.

The vibrational mode from the CeO_2_(110)–R­{111} reconstruction aligns with the observed 2154 cm^–1^ peak, but this surface feature likely forms at edge sites, which are expected to be a relatively minor portion of the nanocube surface. It therefore cannot explain the significantly higher intensity observed in the nanocube sample. We therefore propose that the increased intensity at 2154 cm^–1^ in the nanocube sample primarily arises from CeO_2_(100)-Ce25% surface reconstructions, which yield a strong vibrational mode in this range despite originating from nominally (100) facets. This interpretation is further supported by high-resolution STEM-HAADF images, which reveal single-atom contrast features on the (100) surfaces of the nanocubes (highlighted by white arrows in Figure 3), consistent with the presence of Ce-terminated pyramidal structures.

An additional insight emerges from comparing theoretical and experimental frequencies: the 2128 cm^–1^ mode calculated for structure **3** ((100)-Ce50%) may correspond to the previously unassigned band observed at 2123 cm^–1^ in the DRIFTS spectra (Figure 2). The relatively low intensity of this peak suggests that the CeO_2_(100)-Ce50% reconstruction is less abundant, consistent with previous reports that it is thermodynamically less stable than the CeO_2_(100)-Ce25% configuration.^33,34^

### Cu/CeO_2_ Characterization

With both ceria nanoshapes thoroughly characterized, we next turned to the investigation of the three copper-loaded samples: 1Cu/CeO_2_–NS, 1Cu/CeO_2_–NC, and 0.16Cu/CeO_2_–NC. General characterization, including BET surface area measurements, XRD, Raman spectroscopy, H_2_-TPR, and XPS, has been previously reported and is summarized in the Supporting Information (Table S2).^21,22^ However, although these techniques provide insight into the overall structure and composition, they offer limited detail regarding the specific nature and distribution of the copper species. Previous analysis of the 1% Cu samples by TEM did not provide any information about the copper.^21^ Notably, to the best of our knowledge, in the scientific literature, information on dispersed copper species for ceria-supported copper samples in transmission has been obtained only through high-resolution electron microscopy and only for prereduced samples.^4^ STEM-HAADF also exhibits important difficulties for the detection and analysis of copper in this type of samples although important details could be achieved through the XEDS analytical application.^47^

On that basis, high-yield XEDS mapping (Figure 5) was used to examine the distribution of copper, revealing generally good dispersion across all samples and confirming the presence of subnanometric copper species. However, copper dispersion appears heterogeneous in any case and even more so in the nanocube-supported samples, where localized accumulation of the copper—including the formation of larger particles—was observed between CeO_2_ cubes. This enrichment effect was evident in both 0.16Cu/CeO_2_–NC and 1Cu/CeO_2_–NC samples and was more pronounced in the latter, consistent with the reduced dispersion typically seen at higher metal loadings.

**Figure 5:**
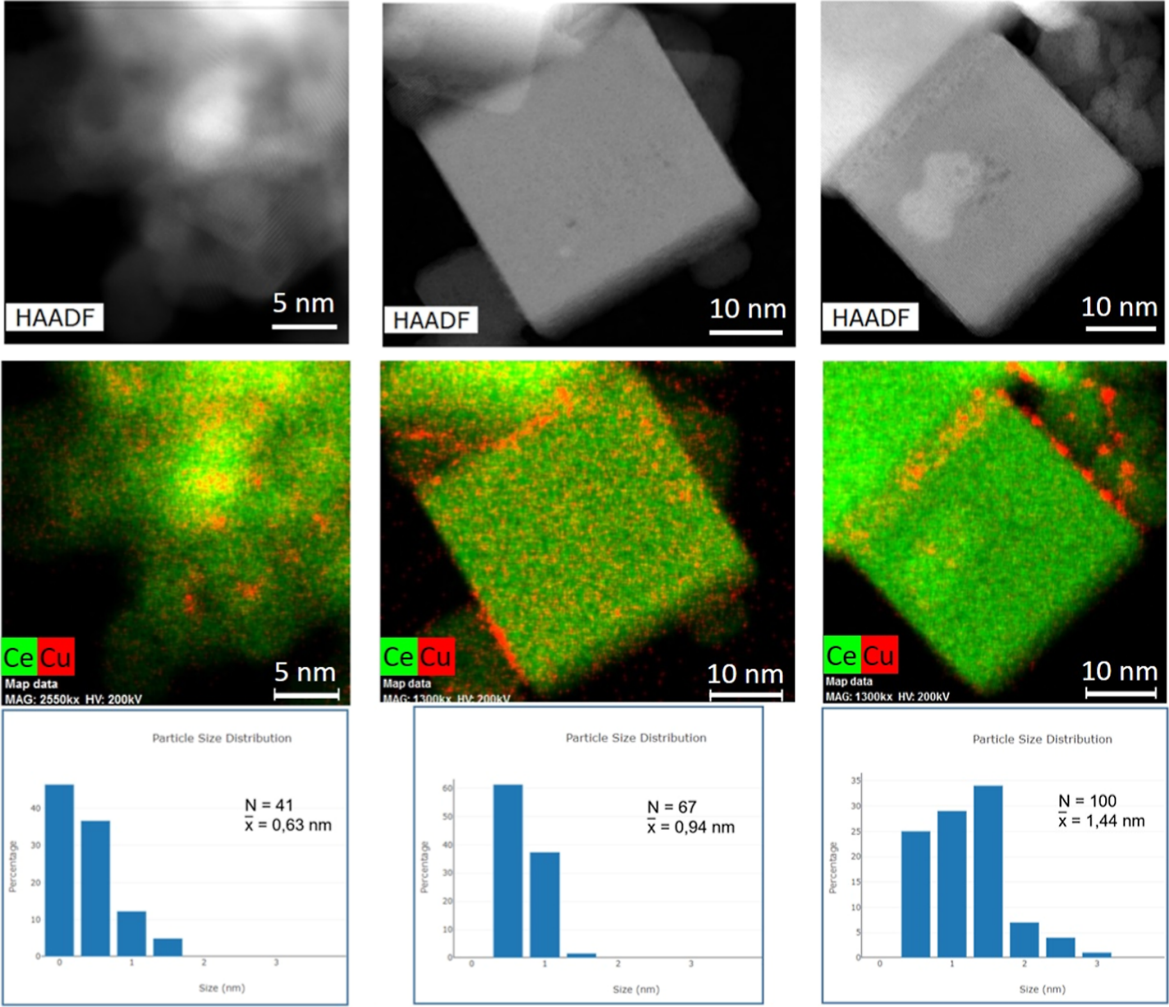
STEM-HAADF images (top row), Ce and Cu distribution XEDS maps (middle row), and particle size distribution (bottom row) for samples 1Cu/CeO_2_-NS (left column), 0.16Cu/CeO_2_-NC (middle column), and 1Cu/CeO_2_-NC (right column). The histograms report the number of particles analyzed (*N*) for each sample, and the average particle diameter (*x̅*) is indicated in each case.

This phenomenon likely originates during the final stages of the impregnation process, where solvent evaporation becomes localized in the interstitial voids between particles, which constitute the pores of the sample. As a result, copper precursors concentrate in these regions at the final stages of solvent evaporation, ultimately leading to higher copper accumulation between adjacent cubes. Nonetheless, smaller copper species were also found to be well dispersed across the terraces of the nanocubes (see Supporting Information, Figure S4).

Figure 5 presents the particle size distributions of copper aggregates derived from multiple XEDS maps for each sample. The number of particles analyzed (**N**) is indicated in each histogram. These values are relatively low due to the extremely small particle sizes, which made identification challenging and required extended acquisition times at high magnification. The smallest and most difficult-to-detect copper species were observed in the 1Cu/CeO_2_–NS sample, followed by 0.16Cu/CeO_2_–NC, while the largest copper nanoparticles were found in 1Cu/CeO_2_–NC. As mentioned, differences in the copper distributions most likely arise from differences in the textural properties of the CeO_2_ nanocubes in comparison with the nanospheres, which modify the dynamics of solvent evaporation during the impregnation process. Table S1 shows the important difference in *S*_BET_ and average pore size, while Figure S5 includes corresponding isotherms as well as the Barrett–Joyner–Halenda (BJH) desorption pore size distribution curves for the two supports and which illustrate such differences.

Further characterization of the copper species in the initial calcined samples was performed by using EPR spectroscopy (Figure 6), complementing the XEDS findings. The spectra confirm high dispersion of copper species in the nanosphere-supported sample, while the nanocube-supported samples contain more concentrated and larger copper particles. All spectra are dominated by Cu^2+^ signals. Additionally, samples supported on nanocubes exhibit an extra axial signal at *g*_⊥_ = 1.967, which is characteristic of CeO_2_ and is commonly attributed to Ce^3+^ ions in specific symmetry environments or to electrons trapped in oxygen vacancies.^48,49^

**Figure 6:**
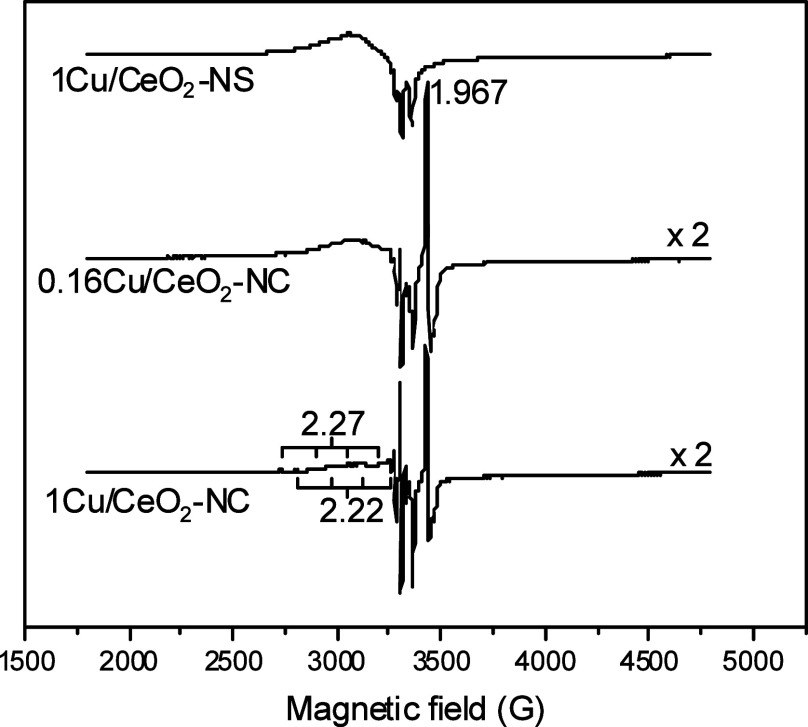
EPR spectra at 100 K for indicated samples.

At *g* > 2, a resolved hyperfine structure is observed, indicating the presence of at least two distinct Cu^2+^ species with g_∥_ = 2.22 and 2.27, respectively. These isolated Cu^2+^ signals exhibit similar g values across all three samples. However, broader linewidths are observed in the 1Cu/CeO_2_–NS sample, which may reflect a wider distribution of local environments or subtle differences in copper–support interactions—consistent with the higher dispersion of copper and the mixed-facet character of the nanosphere support.

Superimposed on these isolated Cu^2+^ features is a broad, structureless, asymmetric signal with edges at approximately *g* ≈ 2.20 and *g* ≈ 2.04 and an average g-value of ⟨*g*⟩ ≈ 2.11. This broad component is attributed to Cu^2+^ species within CuO_*x*_ clusters.^12^ Its relative intensity is highest in the 1Cu/CeO_2_–NS sample, followed by the 0.16Cu/CeO_2_–NC sample, and nearly absent in the 1Cu/CeO_2_–NC sample. Notably, the spectra of the first two samples are similar in this region, reflecting their comparable surface copper densities. In contrast, the weak CuO_*x*_ signal in the 1Cu/CeO_2_–NC sample correlates with more prominent signals from isolated Cu^2+^ species.

This trend is in line with the XEDS results (Figure 5), which indicate a lower copper dispersion and larger particle sizes in the 1Cu/CeO_2_–NC sample. Larger CuO_*x*_ aggregates likely promote stronger antiferromagnetic coupling between Cu^2+^ ions, rendering many of them EPR-silent. Previous XPS studies confirmed that the majority of copper in these preoxidized samples is in the Cu^2+^ state,^12,50^ suggesting that the EPR-inactive fraction primarily corresponds to CuO-type nanoparticles exhibiting strong antiferromagnetic interactions. This interpretation is supported by quantitative EPR analysis, which shows that 59% of the total copper is EPR-active in the 1Cu/CeO_2_–NS sample, compared to only 14% in 0.16Cu/CeO_2_–NC and 4% in 1Cu/CeO_2_–NC. These results suggest that a large proportion of copper in all samples remains EPR-silent, most likely due to the presence of mainly antiferromagnetically coupled Cu^2+^ ions, as typically found in bulk CuO.^51^

As with the unloaded supports, the surface properties of the copper-loaded samples in their calcined state were further investigated by using low-temperature DRIFTS with CO as a probe molecule (Figure 7). This analysis revealed the formation of carbonyl species adsorbed on both ceria and copper sites, with bands attributed to copper-bound CO appearing below approximately 2130 cm^–1^.

**Figure 7:**
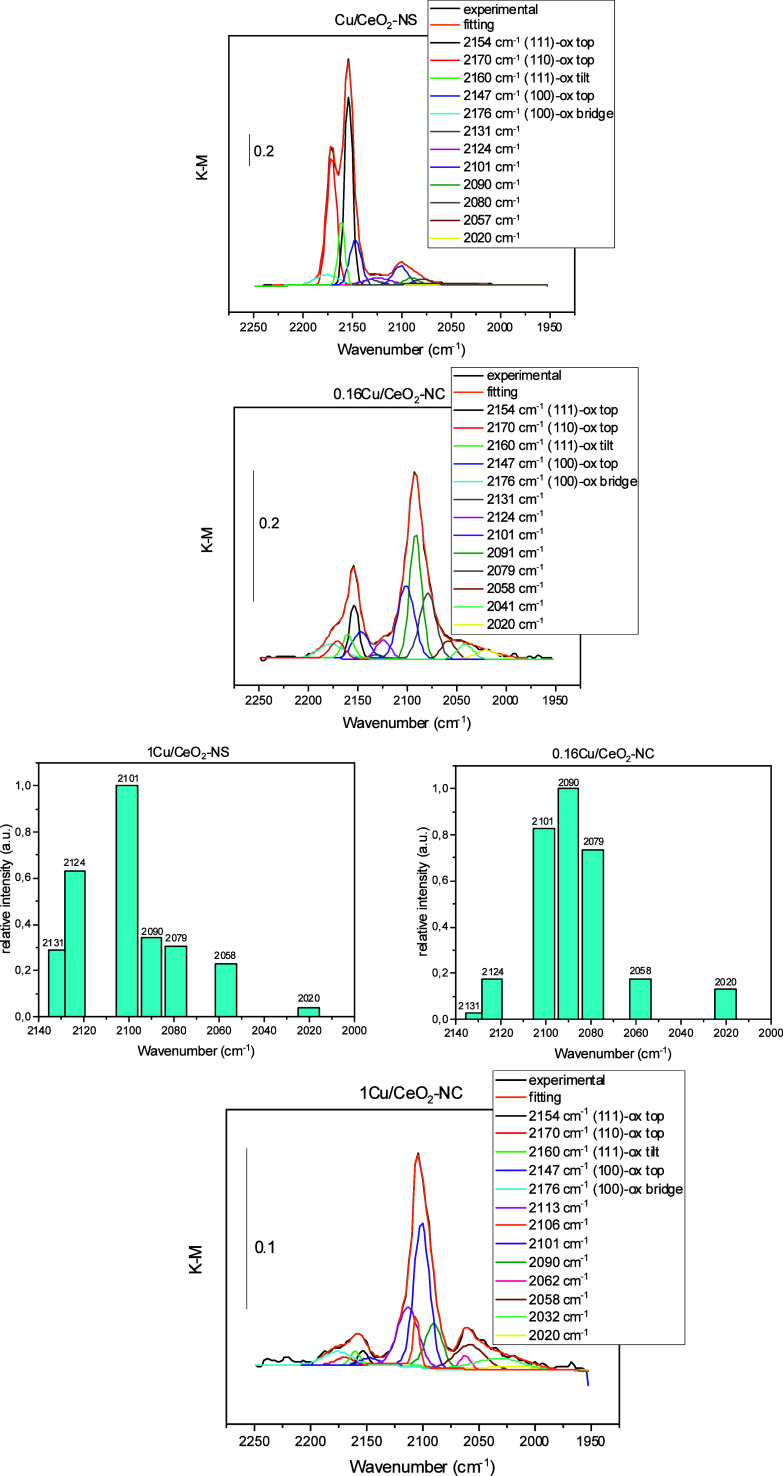
DRIFTS spectra recorded under 1% CO/N_2_ flow at 137 K over Cu-CeO_2_-NS (top), 0.16Cu-CeO_2_-NC (middle), and 1Cu/CeO_2_-NC (bottom), pretreated under 10% O_2_/N_2_ at 653 K and subsequently cooled under N_2_ flow. The figure also shows the comparison of normalized CO vibrational bands for frequencies associated with CO adsorbed on Cu species in the 1Cu/CeO_2_–NS and 0.16Cu/CeO_2_–NC samples. Bars represent relative intensities extracted from the DRIFTS spectra, normalized to the most intense Cu-related feature in each sample.

Notably, the ratio between the intensities of the carbonyl bands associated with copper and those associated with the support is higher for the nanocube-supported samples than for those supported on nanospheres. Two main factors may contribute to this trend. First, copper deposition can partially cover the support surface, as supported by S_BET_ measurements (Tables S1 and S2), reducing the number of accessible ceria sites. This effect was previously observed via XPS, which showed a diminished cerium signal in the 1Cu/CeO_2_–NC sample, attributed to surface coverage by relatively large copper particles.^21^ A similar pattern emerges in the DRIFTS spectra: 1Cu/CeO_2_–NC shows a marked decrease in the support-associated carbonyl intensity compared to 0.16Cu/CeO_2_–NC.

Second, the nature and redox state of the surface copper species strongly influence the intensity of the copper-associated carbonyl bands. Carbonyls adsorbed on Cu^2+^ sites are typically weakly bound and detectable only at temperatures below ∼130 K. Therefore, the observed intensity of these bands depends on the degree of surface copper reduction prior to CO exposure—a process influenced by both particle size and copper–ceria interaction, with smaller particles generally being more readily reduced.^21,52–54^ However, since most of the copper in the calcined and preoxidized samples is expected to remain in the +2 oxidation state,^12,50^ it is likely that only a small fraction of the total copper contributes to the observed carbonyl features.

Note that, from the DRIFTS spectra alone, the presence of CO features corresponding to Cu^2+^ adsorption sites cannot be completely ruled out, since they would fall in the region of spectra discussed for clean ceria surfaces (>2140 cm^–1^). Given that, as just mentioned, we have no clear basis to quantitatively compare intensities or otherwise discern between the two, we focused on the study of the Cu^+/0^ region.

To support the interpretation of the DRIFTS spectra, we modeled both single Cu atoms (Cu_1_) and Cu_4_ clusters adsorbed on a range of unreconstructed and reconstructed ceria surfaces (Figures S6 and S7). CO molecules were then adsorbed onto these models, and their vibrational frequencies were calculated using the HSE06 functional for improved accuracy. The surfaces considered include CeO_2_(111), (110), (100)-O, the reconstructed (110)-R{111}, and (100)-Ce_25%_ facets, as described earlier. Additionally, a stepped model was generated by intersecting two (100)-O planes to simulate the site formed at the junction between two neighboring nanocubes (denoted as (100)-O//(100)-O), where copper accumulation due to solvent evaporation had been observed (Figure S7).

On all surfaces, Cu_1_ becomes oxidized to Cu^+^ upon adsorption, with Bader charges of approximately +0.7 e^–^, and a single Ce^3+^ center forms on the ceria support (Figure 8a and Table 3). In the case of Cu_4_ clusters, two electrons are transferred to the support, resulting in a total Bader charge of 1.5–1.8 e^–^ and the formation of two Ce^3+^ sites (Figure 8b and Table 3).

**Figure 8:**
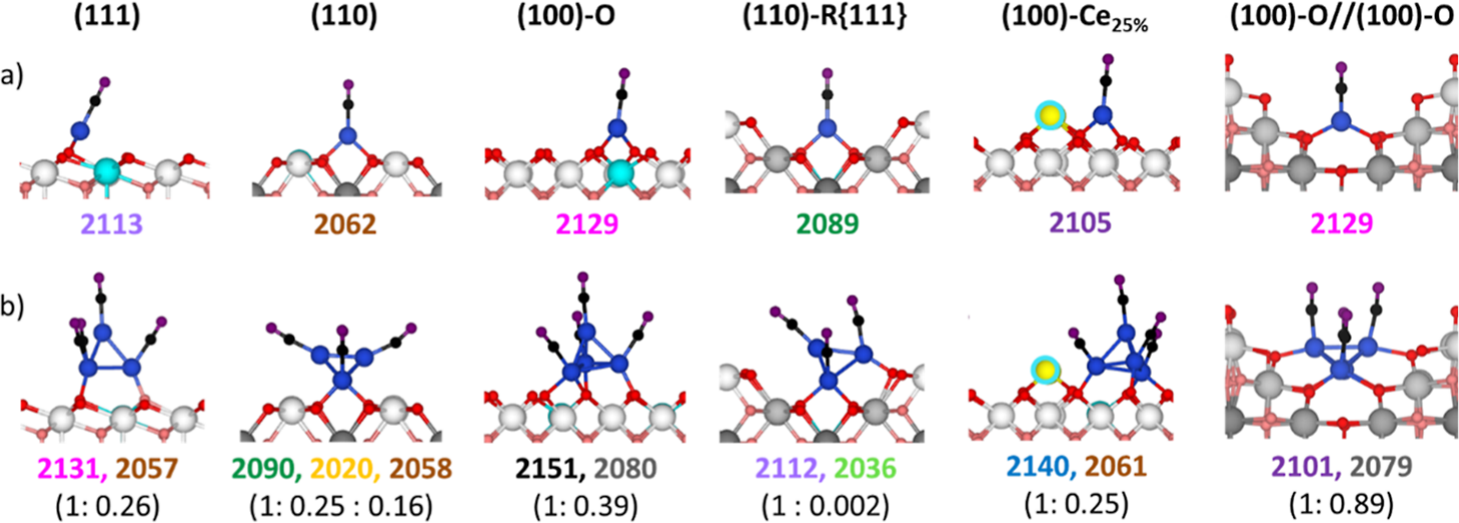
Side views for the optimized structures for CO adsorption on (a) a single Cu atom (Cu_1_) and (b) a Cu_4_ cluster on various ceria surfaces. CO vibrational frequencies calculated at the HSE06 level are shown in cm^–1^, with colors corresponding to the experimental bands in Figure 7. Relative intensities are also provided. Surface Ce and O atoms are shown in white and red, respectively; subsurface Ce and O atoms are rendered in gray and pink. Carbon and oxygen atoms from adsorbed CO are shown in black and purple, respectively. Ce atoms forming pyramidal reconstructions are highlighted in yellow, Cu atoms in dark blue, and Ce^3+^ sites in light blue. See also Figure S8 for top views.

**Table 3:** Calculated Properties for Cu_1_ on Various Ceria Surfaces, as Shown in Figure 8 ^,^ ^a^

Surface	(111)	(110)	(100)-O	(110)-R{111}	(100)-Ce_25%_	(100)-O//(100)-O
Δ*E*(Cu_1_)_b_	–1.86	–3.27	–3.48	–2.60	–3.55	–4.01
Δ*E*(CO)_ads_	–1.23	–0.78	–0.67	–1.39	–0.43	–0.43
ν_CO_	2113	2062	2129	2089	2105	2129
*q*_Cu1_	0.72	0.74	0.73	0.71	0.74	0.76

a Reported values include copper binding energy (Δ*E*(Cu_1_)_b_), CO adsorption energy (Δ*E*(CO)_ads_) in eV, CO vibrational frequency (ν_CO_) in cm^–1^, and Bader charge on Cu (*q*_Cu1_) in e^–^.

The binding energy for both single Cu atoms and Cu_4_ clusters is highest on the (100)-O surface, its reconstructed (100)-Ce_25%_ form, and the stepped (100)-O//(100)-O configuration (Tables 3 and 4). This trend is consistent with STEM-HAADF observations, which show that small, well-dispersed copper species preferentially localize on the (100) facets of nanocubes—particularly on open surfaces not involved in interparticle contact.

**Table 4:** Calculated Properties for Cu_4_ on Various Ceria Surfaces, as Shown in Figure 8 ^a^

surface	(111)	(110)	(100)-O	(110)-R{111}	(100)-Ce_25%_	(100)-O//(100)-O
Δ*E*(Cu_4_)_b_	–2.35	–2.37	–2.77	–2.66	–2.69	–3.11
Δ*E*(CO)_ads_	–0.66	–0.77	–0.71	–0.67	–0.71	–0.38
ν_CO_	2131, 2057	2090, 2020, 2058	2151, 2080	2112, 2036	2140, 2061	2101, 2079
q_Cu4_	1.57	1.48	1.70	1.84	1.74	1.79

a Reported values include copper binding energy (Δ*E*(Cu_4_)_b_), CO adsorption energy (Δ*E*(CO)_ads_) in eV, CO vibrational frequency (ν_CO_) in cm^–1^, and sum of Bader charges on Cu atoms in Cu_4_ (*q*_Cu_4__) in e^–^.

The stepped (100)-O//(100)-O model was constructed to mimic the interface formed at the junction between two nanocubes, where copper accumulation is experimentally observed. The strong binding calculated for this stepped configuration helps to explain the stabilization of copper at these junctions. However, it is important to emphasize that the formation of large copper particles in these regions is not solely driven by surface thermodynamics. Instead, it can primarily result from localized solvent evaporation during the final stages of impregnation, which concentrates the precursor in confined interparticle pores. Therefore, while the (100)-O//(100)-O site is indeed a favorable binding site, its role in sintering is largely dictated by the local environment created during synthesis rather than by any intrinsic tendency of the (100) surface to promote copper aggregation.

In contrast, the (111) surface shows the weakest copper binding energy and is not expected to stabilize small oxidized copper species as effectively. Such types of surfaces could be the location of larger CuO-type particles, as observed by EPR, in the nanosphere (NS) sample, where such an open (111) surface is present. In any case, for the nanocubes, the textural properties of the nanospheres most likely determine the copper distribution over the surface. In this case, a more uniform wetting of the surface during impregnation and subsequent evaporation of the solvent could be most relevant to explain the higher copper dispersion inferred from XEDS and EPR results (Figures 5 and 6).

When focusing on the vibrational bands associated with CO adsorption on copper sites (i.e., excluding those related to ceria), the best agreement between the DRIFTS spectra in Figure 7 and the theoretical models developed in this work is found for the samples containing smaller copper particles—namely, 1Cu/CeO_2_–NS and 0.16Cu/CeO_2_–NC. The calculated vibrational frequencies for Cu_1_ on the reconstructed (110)-R{111} (2089 cm^–1^, Figure 8a and Table 3) and (100)-Ce_25%_ (2105 cm^–1^) surfaces align closely with the two most intense experimental features observed in the 0.16Cu/CeO_2_–NC sample (2090 and 2101 cm^–1^, Figure 7). In contrast, while both bands are present in the 1Cu/CeO_2_–NS spectrum, the 2090 cm^–1^ peak is markedly less intense than the 2101 cm^–1^ one. This suggests a more significant contribution from Cu atoms on (100)-Ce_25%_ (2105 cm^–1^) surfaces in both samples, whereas Cu on (110)-R{111} surfaces appears to be less abundant in the NS system, in agreement with TEM exploration (Figure 1).

Clusters on the (110) and (100)-O//(100)-O surfaces can contribute to the experimental bands at 2090 and 2101 cm^–1^, respectively. Their secondary calculated frequencies—2020 and 2058 cm^–1^ for (110), and 2079 cm^–1^ for (100)-O//(100)-O (Table 4)—are also observed in the DRIFTS spectra and appear more prominently in the nanocube sample, suggesting a lower abundance of these Cu_4_ clusters in the nanosphere system. Notably, the calculated 2079 cm^–1^ band (corresponding to the experimental 2080 cm^–1^ feature) is significantly more intense in the nanocube spectrum. This observation supports its assignment to Cu_4_ clusters on (100)-O//(100)-O or (100)-O surfaces, both of which are more structurally accessible in nanocube morphology.

This interpretation is further reinforced by the co-observation of the strongest calculated modes associated with these clusters: 2101 cm^–1^ for Cu_4_ on (100)-O//(100)-O and 2151 cm^–1^ for Cu_4_ on (100)-O. Although the latter is likely obscured by the nearby intense ceria band at 2154 cm^–1^, its presence cannot be ruled out. For Cu_4_ clusters on (110), the dominant calculated frequency at 2090 cm^–1^ is also present in the spectra, along with its weaker satellite modes at 2058 and 2020 cm^–1^—forming a consistent and distinguishable fingerprint.

The three dominant Cu-derived carbonyl bands observed in the DRIFTS spectra—at approximately 2090, 2101, and 2080 cm^–1^—are well reproduced by the theoretical models developed in this work. Specifically, the 2090 cm^–1^ band corresponds to Cu_1_ on (110)-R{111} and Cu_4_ on (110) surfaces; the 2101 cm^–1^ band matches Cu_1_ on (100)-Ce_25%_ and Cu_4_ on (100)-O//(100)-O; and the 2080 cm^–1^ feature is attributed to Cu_4_ clusters on (100)-O//(100)-O and (100)-O surfaces.

Altogether, the simultaneous presence of both high-intensity and satellite vibrational features strongly supports the existence of small copper clusters stabilized on the (100)-O//(100)-O, (100)-O, and (110) ceria surfaces. This agreement between theory and experiment reinforces the atomistic models proposed and underscores the ability of vibrational spectroscopy, when paired with detailed surface modeling, to resolve subtle structural differences among supported copper species (Figure 8b and Table 4). This nuanced frequency–structure relationship contrasts with classical interpretations, which often relied on broad correlations between CO stretching frequencies and copper oxidation states, without resolving the structural origin of spectral features.^55,56^

In addition to the three most prominent signals discussed above (2090, 2101, and 2080 cm^–1^), several additional vibrational bands are also consistently observed in both samples, namely, at 2131, 2124, and 2058 cm^–1^ (Figure 7). The 2124 cm^–1^ band, also observed in the spectrum of unloaded ceria (cf. 2123 cm^–1^ in Figure 2), can be assigned to CO adsorption on CeO_2_(100)-Ce_50%_ surfaces (cf. 2128 cm^–1^ in Figure 4). A slight increase in intensity in the nanocube sample may suggest an additional contribution from copper species, particularly from Cu atoms on (100)-O or (100)-O//(100)-O facets, for which calculated frequencies of 2129 cm^–1^ are found (Figure 8a and Table 3).

Similarly, the 2058 cm^–1^ band in the nanocube sample is too intense to be attributed solely to the weaker vibrational modes of Cu_4_ clusters on CeO_2_(110) or (100)-Ce_25%_ surfaces (cf. 2058 and 2061 cm^–1^ in Figure 8b, Table 4). This suggests a contribution from clusters of different sizes and geometries, but it could also come from mononuclear copper species on CeO_2_(110), consistent with the calculated frequency at 2062 cm^–1^ (Figure 8a, Table 3). The lower intensities of the 2124 and 2058 cm^–1^ bands in the nanosphere sample further support their assignment to copper species located on (100)- and (110)-derived surfaces, which are more abundant in the nanocube morphology. The 2131 cm^–1^ band may be assigned to Cu_4_ clusters on CeO_2_(111) surfaces (Figure 8b and Table 4). In contrast, the calculated 2113 cm^–1^ mode—also associated with the (111) surface—is not observed in either sample, likely due to the lower binding energy of copper on this facet and its correspondingly lower stability.

A broad band at 2041 cm^–1^ can be present in both samples but appears clearly more intense and in the 0.16Cu/CeO_2_–NC sample. Although this feature cannot be confidently assigned within the current theoretical models while it is present in the copper-free support and related to CO adsorbed on support defects (Figure 2), we cannot discard an enhanced intensity, which may indicate contributions from Cu species of different nuclearity or coordination environments, potentially associated with the lower oxidation state and greater structural diversity of copper on this morphology.

For the higher-loaded 1Cu/CeO_2_–NC sample, vibrational bands common to the previously discussed systems are again observed, namely, at 2101 cm^–1^ (Cu_1_/CeO_2_(100)-Ce_25%_ and Cu_4_/CeO_2_(100)-O//(100)-O, Figure 8 and Tables 3 and 4), 2090 cm^–1^ (Cu_1_/CeO_2_(110)-R­{111} and Cu_4_/CeO_2_(110)), 2058 cm^–1^ (Cu_1_/CeO_2_(110), and less intense Cu_4_/CeO_2_(110) and Cu_4_/(100)-Ce_25%_), and 2020 cm^–1^ (Cu_4_/CeO_2_(110), Figure 8b and Table 4).

However, a detailed fitting reveals additional bands at 2113, 2106, 2062, and 2032 cm^–1^. As discussed for the 2040 cm^–1^ feature in the 0.16Cu/CeO_2_–NC sample, the broader 2062 and 2032 cm^–1^ bands likely originate from copper clusters of different nuclearity, indicative of a distribution of species beyond those described by the current models. The sharper 2113 cm^–1^ signal can be attributed to Cu_4_ clusters at the reconstructed CeO_2_(110)-R­{111} surface (cf. 2112 cm^–1^, Figure 8b and Table 4). This assignment is further supported by the decreased relative intensity of the 2090 cm^–1^ band associated with isolated Cu atoms on the same facet, suggesting the growth of larger copper species, in line with structural characterization data. Finally, the 2106 cm^–1^ band—unaccounted for by the present theoretical models—has been previously reported for copper nanoparticles,^57^ which are indeed observed in this sample (Figure 5). It is thus not possible to fully reproduce the experimental spectrum of the higher-loaded 1Cu/CeO_2_–NC sample using only the copper species included in the current theoretical framework. This limitation likely reflects the coexistence of copper clusters with a wider distribution of atomicities, in agreement with the XEDS results (Figure 5). Nevertheless, the relative increase in spectral intensity below 2070 cm^–1^, compared to the 0.16Cu/CeO_2_–NC sample, supports a greater contribution from copper clusters over single atoms—consistent with the larger average copper particle size inferred from XEDS and EPR characterization (Figures 5 and 6).

In summary, the most intense vibrational features in all samples—2090 and 2101 cm^–1^—are consistent with the presence of small copper species on CeO_2_(110)-R­{111} and (100)-Ce25% facets and appear more prominently in the nanocube samples. The 2106 cm^–1^ feature observed in the 1Cu/CeO_2_–NC sample is attributed to copper nanoparticles, which were not included in the present modeling. Likewise, while broad bands around 2040 cm^–1^ remain unassigned, the strong agreement between experiment and theory for the less common features at ∼2060 and ∼2020 cm^–1^ supports their assignment to small copper species. Therefore, the remaining unassigned low-frequency bands likely originate from copper clusters of different atomicity than those explicitly modeled, underscoring the diversity of supported Cu species and the sensitivity of vibrational spectroscopy—when paired with atomic-level modeling—to distinguish among them. These insights not only validate the predictive value of the theoretical models employed here but also provide a foundation for future catalyst design strategies targeting structure-sensitive reactions over atomically dispersed and subnanometer copper species.

### Catalytic Activity

Figure 9 presents the catalytic performance of the three samples under the CO-PROX conditions. Among them, 1Cu/CeO_2_–NS exhibits the highest CO oxidation activity, though it also shows the lowest selectivity toward CO_2_ (understood as the portion of O_2_ which reacts selectively with CO in comparison with that reacting with H_2_, which are the two main competing reactions). This is evidenced by a decline in the level of CO conversion following its initial peak, likely due to a higher rate of H_2_ oxidation competing for surface sites. In contrast, the nanocube-supported samples exhibit lower CO oxidation activity, which further diminishes with decreasing copper loading. However, these nanocube systems demonstrate markedly enhanced selectivity toward CO_2_, maintaining complete CO conversion over a broader temperature window. This behavior reflects a suppression of H_2_ oxidation relative to that of CO oxidation, which is critical for effective CO-PROX operation.

**Figure 9:**
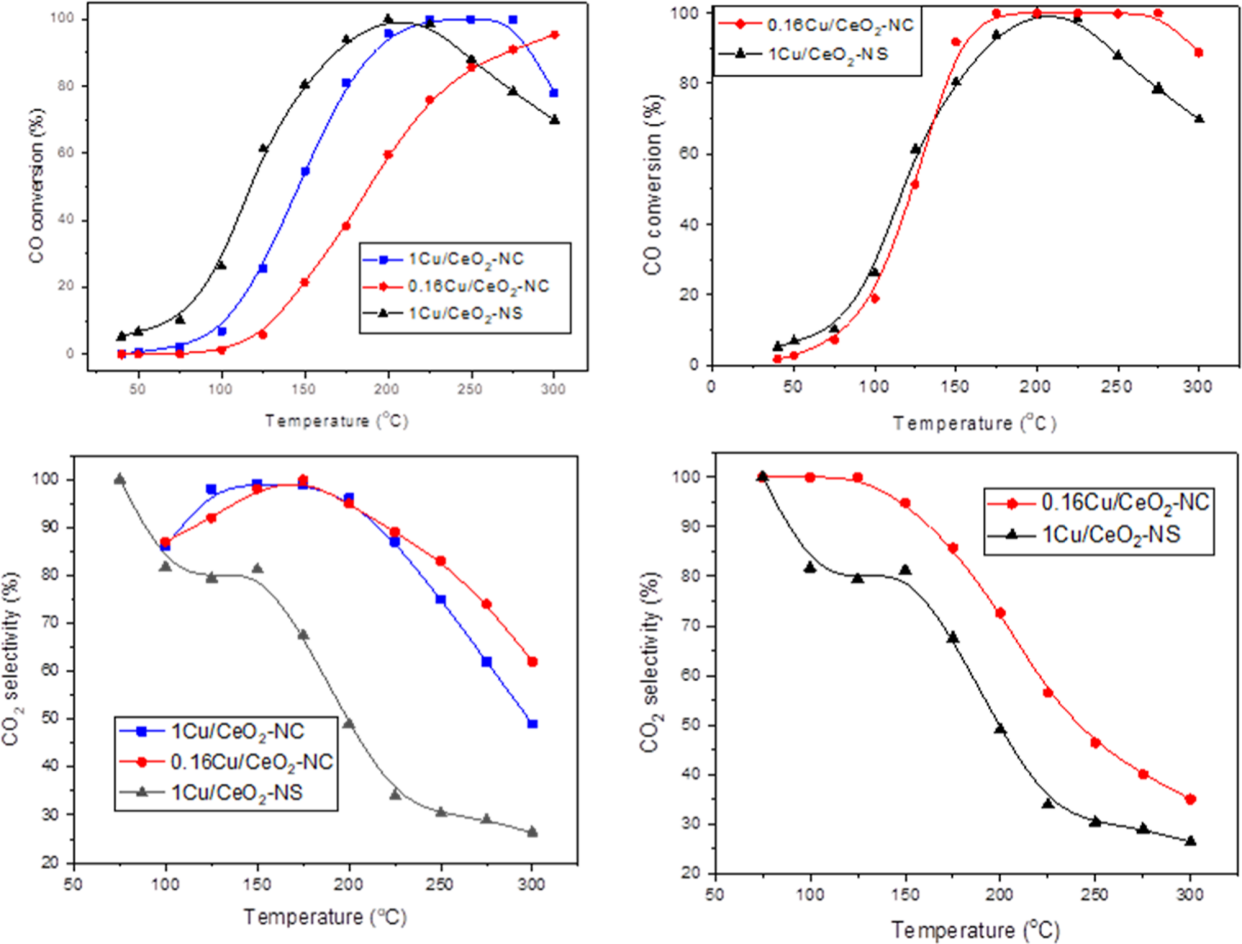
CO conversion and CO_2_ selectivity under 1% CO + 1.25% O_2_ + 50% H_2_ in He, 100 mL min^–1^, 100 mg sample (left), and 100 mg 1Cu/CeO_2_–NS vs 625 mg 0.16Cu/CeO_2_–NC (right).

To decouple the effects of morphology and copper content, a complementary experiment compared the performance of 1Cu/CeO_2_–NS and 0.16Cu/CeO_2_–NC under identical total copper amounts but different space velocities (Figure 9, right). A substantial increase in the residence time over that of 0.16Cu/CeO_2_–NC produces an important increase in its CO conversion. Nevertheless, while 1Cu/CeO_2_–NS continues to display higher CO conversion, 0.16Cu/CeO_2_–NC offers substantially higher CO_2_ selectivity, reinforcing the importance of the morphology and surface structure. The activity of this type of catalysts has been related to the formation of partially reduced interfaces upon interaction of the catalyst with the CO-PROX mixture.^7,11,12^ According to the activity results, different activities are produced as a function of the support in each case which evidence differences in the nanomorphology of the interfacial sites formed and also in agreement with differences in apparent activation energies observed, as examined in a previous work.^21^ Operando DRIFTS previously revealed that carbonyl species on nanocube-supported samples are more stable than those on nanosphere-supported analogues.^21^ This enhanced stability implies a stronger or more selective CO adsorption on specific Cu sites in the nanocube catalysts, which are less prone to activation by H_2_, thereby delaying or suppressing H_2_ oxidation.

This finding is consistent with earlier characterizations, which suggest two contributing factors. First, as the copper particle size increases across the series 1Cu/CeO_2_–NS → 0.16Cu/CeO_2_–NC → 1Cu/CeO_2_–NC, the reducibility of small clusters with higher atomicity decreases sharply.^53,54^ In particular, larger clusters may undergo irreversible oxidation under the reaction conditions, thereby suppressing mostly H_2_ oxidation.

Second, the enhanced CO selectivity observed for the 0.16Cu/CeO_2_–NC sample correlates with its greater abundance of Cu species stabilized on (100)-derived ceria surfaces—such as (100)-Ce25% and (100)-O//(100)-O—identified by their characteristic vibrational frequencies at 2101 and 2080 cm^–1^, respectively. On these facets, the binding of Cu atoms is calculated to be stronger than that on (111) or (110)-derived surfaces, which may help to stabilize isolated Cu species and modulate their interaction with reactants. In contrast, the higher H_2_ oxidation activity observed for 1Cu/CeO_2_–NS is consistent with the prevalence of Cu species on (110)-R{111} or (111)-like surfaces, where CO adsorption energies are higher and carbonyl species are less strongly stabilized, allowing for a different competition between CO and H_2_ activation.

The trend in catalytic stability also aligns with this picture: 1Cu/CeO_2_–NS may deactivate due to weaker Cu–support binding on (111) facets (Table 3), while 1Cu/CeO_2_–NC—despite its higher selectivity—may suffer from sintering at increased copper loading relative to 0.16Cu/CeO_2_–NC. Overall, these observations emphasize the dual role of facet-dependent copper speciation and particle nuclearity in governing both selectivity and durability under CO-PROX conditions.

Reconstruction of CeO_2_ nanocubes involves the thermal transformation of the (110) edge facets into (111) sawtooth-like zigzag geometries, as observed here and previously reported in the literature.^14,17,44,46^ On the other hand, the (100) facets of the nanocubes are polar and intrinsically unstable and therefore undergo reconstruction to compensate for the surface dipole.^20,34,58^ This reconstruction may involve half-termination with oxygen or cerium atoms, or termination by CeO_4_ pyramids.^20,34,58^ Thermal reconstruction toward the more stable (111) surfaces typically leads to a decrease in the catalytic CO oxidation activity of the CeO_2_ supports. This effect has generally been attributed to a lower concentration of defective Ce^3+^–oxygen vacancy sites on the (111) surface compared with the (100) surface.^14,46^ For CeO_2_-supported metal catalysts, however, the situation is more complex due to the heterogeneity of possible interfacial active sites. For instance, reconstruction of nanocube edges has been reported to enhance CO oxidation activity in supported gold catalysts.^16^ Similarly, interfaces between palladium and CeO_2_ (111) surfaces appear more active for CO oxidation than those formed on CeO_2_ (100) surfaces.^59^ Conversely, other studies have reported a negative effect of nanocube reconstruction on CO oxidation activity—and under CO-PROX conditions—for supported gold catalysts, along with a decrease in CO_2_ selectivity during the CO-PROX process.^60^ In our case, the copper-supported reconstructed nanocubes exhibit interfacial sites that are less active for CO oxidation than those observed over nanospheres. However, these sites display significantly higher selectivity toward the CO_2_ formation.

## Conclusions

In this work, we have shown that ceria nanomorphology decisively governs the structural, electronic, and catalytic properties of low-loaded Cu/CeO_2_ catalysts for CO-PROX. Using advanced microscopy, spectroscopy, and DFT modeling, we directly correlated the support shape with copper speciation and interfacial chemistry. Although both CeO_2_ nanospheres and nanocubes reconstruct extensively toward (111)-like surfaces, nanocubes additionally stabilize reconstructed (100)- and (110)-derived facets. These environments promote stronger CO binding, distinct carbonyl signatures, and heterogeneous Cu distributions with both isolated species and aggregates, whereas nanospheres favor more uniform dispersion and weaker CO stabilization.

These structural distinctions manifest in clear catalytic consequences: nanosphere-supported catalysts exhibit higher CO oxidation activity, while nanocube-supported catalysts achieve superior CO_2_ selectivity and a broader full-conversion window. The combined experimental and theoretical results identify copper speciation and surface carbonyls as key descriptors linking the CeO_2_ morphology to catalytic function. Beyond providing a structure–performance framework for Cu/CeO_2_, this work underscores the broader concept that nanoshape engineering of reducible oxides offers a powerful strategy to tune metal–support interactions and guide the rational design of cost-effective catalysts for selective oxidation reactions.

## Supplementary Material



## Data Availability

The DFT data that support the findings of this study are available in Materials Cloud {https://www.materialscloud.org/home} with the identifier DOI: 10.24435/materialscloud:9m-z3. The data are also available from the authors upon reasonable request.

## Notes

Tables summarizing the basic physicochemical properties of the samples obtained from XRD, Raman, and N_2_ adsorption measurements, as well as computational models describing the different surface configurations and adsorption scenarios considered in this work, additional data showing the variation in the intensity of carbonyl bands observed on CeO_2_–NC as a function of CO adsorption temperature, XEDS maps and corresponding spectra for copper species supported on CeO_2_–NC, together with N_2_ adsorption–desorption isotherms, and BJH desorption pore size distribution plots for the CeO_2_ supports (PDF)
