# Supplementary material for: Fine-Tuning Catalysts: The Role of Support Nanomorphology in Shaping Cu/CeO2 CO-PROX Properties
Source: ACS Catal. 2025 Nov 25;15(24):20360–76. doi: 10.1021/acscatal.5c06552 (PMC12724332; doi:10.1021/acscatal.5c06552)
Supplement: Supplementary file 1 [file cs5c06552_si_001.pdf]

SUPPORTING INFORMATION

Fine-Tuning Catalysts: The Role of Support Nanomorphology in Shaping Cu/CeO_2 CO-PROX Properties

Estefanía Fernández-Villanueva[1,2,⊥], Patricia Pérez-Bailac[1,⊥], Pablo G. Lustemberg[1], Ana B. Hungría[3,*], Laura Pascual[1], Renato Cataluña[4], Jose A. Vidal-Moya[5], Teresa Blasco[5], M. Verónica Ganduglia-Pirovano[1,*], Arturo Martínez-Arias[1,*]

[1]*Instituto de Catálisis y Petroleoquímica. CSIC. C/ Marie Curie 2. 28049 Madrid (Spain).*

[2]*Universitat Politècnica de València, Camí de Vera s/n 46022 (Spain).*

[3]*Departamento de Ciencia de Materiales, Ingeniería Metalúrgica y Química Inorgánica, Facultad de Ciencias, Universidad de Cádiz. 11510 (Spain).*

[4]*Universidade Federal do Rio Grande do Sul, Porto Alegre, RS, BR-91501970 (Brazil).*

[5]*Instituto de Tecnología Química, Universitat Politècnica de València – Consejo Superior de Investigaciones Científicas (UPV-CSIC), 46022 Valencia, Spain*

[⊥] These authors contributed equally.

[*] Corresponding authors. E-mail: ana.hungria@uca.es, vgp@icp.csic.es, amartinez@icp.csic.es.

Table S1. Basic physicochemical properties of the supports as determined by XRD, Raman, and S_{BET} measurements and pore size estimation from the N_2 adsorption isotherms.[1]

Sample	CeO_2 crystallite size (nm)	CeO_2 lattice parameter a (Å)	Microstrain (Δd/d)	F_{2g} frequency (cm^{-1})	F_{2g} FWHM (cm^{-1})	S_{BET} (m^2g^{-1})	Average pore size (nm)
CeO_2-NC	46	5.406	0.00012	463	15.5	20	18
CeO_2-NS	7	5.410	0.0019	462	23.3	130	3

Table S2. Basic physicochemical properties of the indicated catalysts as determined by XRD, Raman, and S_{BET} measurements.[1,2]

Sample	CeO_2 crystallite size (nm)	CeO_2 lattice parameter a (Å)	Microstrain (Δd/d)	F_{2g} frequency (cm^{-1})	F_{2g} FWHM (cm^{-1})	S_{BET} (m^2g^{-1})
1Cu/CeO_2-NC	42	5.404	0.00020	463	15.5	14
0.16Cu/CeO_2-NC	42	5.404	0.00020	463	15.4	16
1Cu/CeO_2-NS	7	5.410	0.0025	460	28.2	115

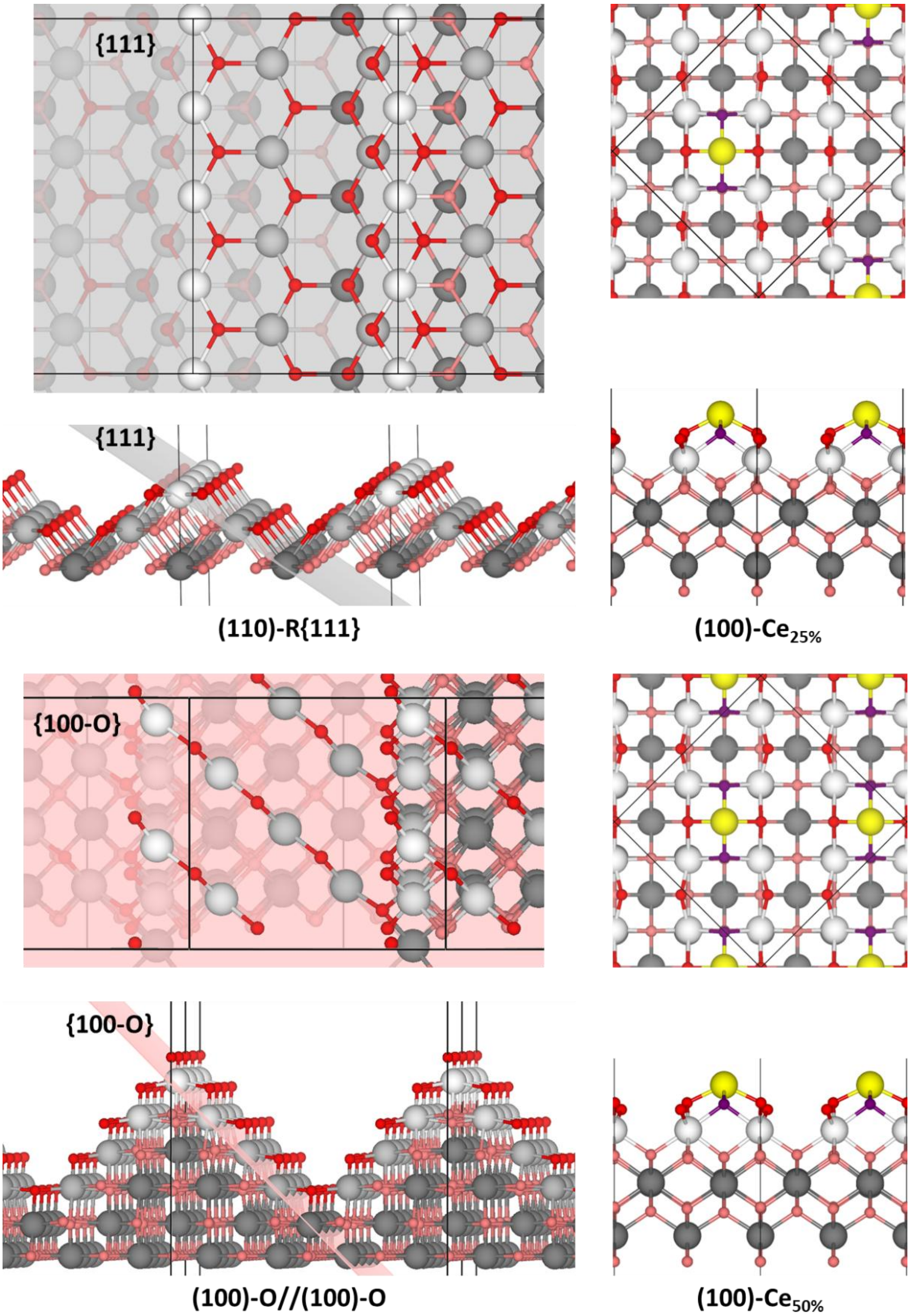

Figure S1. Computational models for the (110)-R{111} and (100)-Ce reconstructed surfaces, as well as the (100)-O//(100)-O model created to model two (100)-O surfaces close together. Surface Ce and O atoms are depicted in white and red, respectively, while deeper Ce and O atoms are shown in grey and pink. Added Ce atoms, forming CeO_4 pyramids, are highlighted in yellow, with their corresponding added two O atoms displayed in purple.

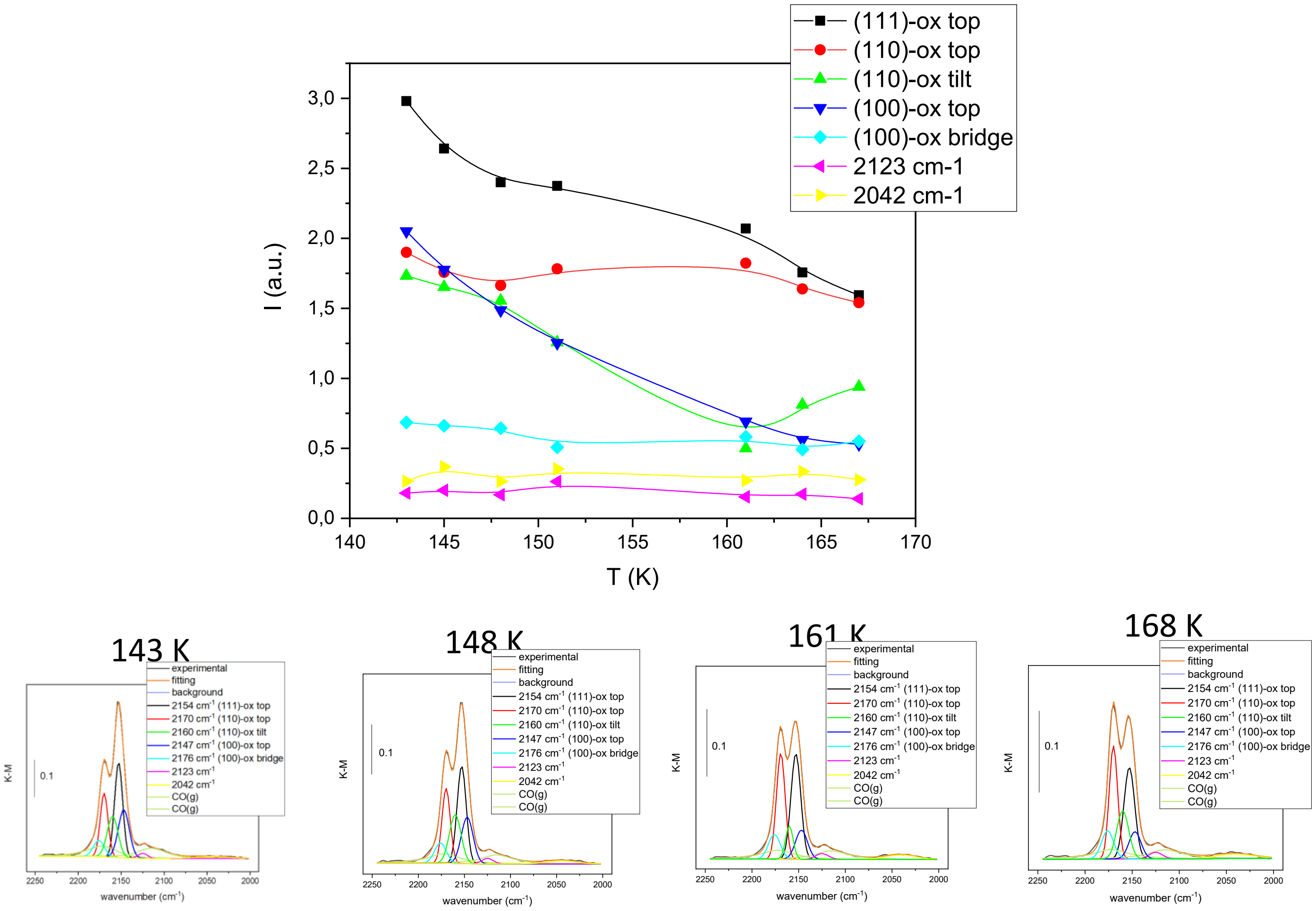

Figure S2. Evolution of the intensity of the indicated carbonyls for CeO_2-NC (pretreated under 10% O_2/N_2 at 653K and cooled under N_2) under 1% CO/N_2 at the indicated temperature. Fittings for selected spectra are shown below.

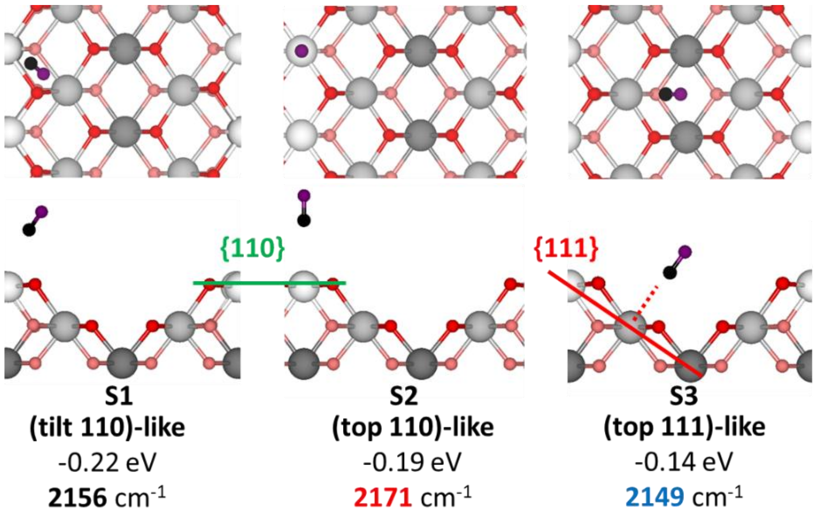

Figure S3. Adsorption structures of a CO molecule (low coverage) on the (110)-R{111} model, calculated at the HSE level. CO adsorption energies and vibrational frequencies are provided. The {111} and {110} planes are indicated in the sawtooth structures, shown in red and green, respectively. Surface Ce and O atoms are depicted in white and red, respectively, while deeper Ce and O atoms are depicted in grey and pink. O atoms from CO molecules are represented in purple, and C atoms are shown in black.

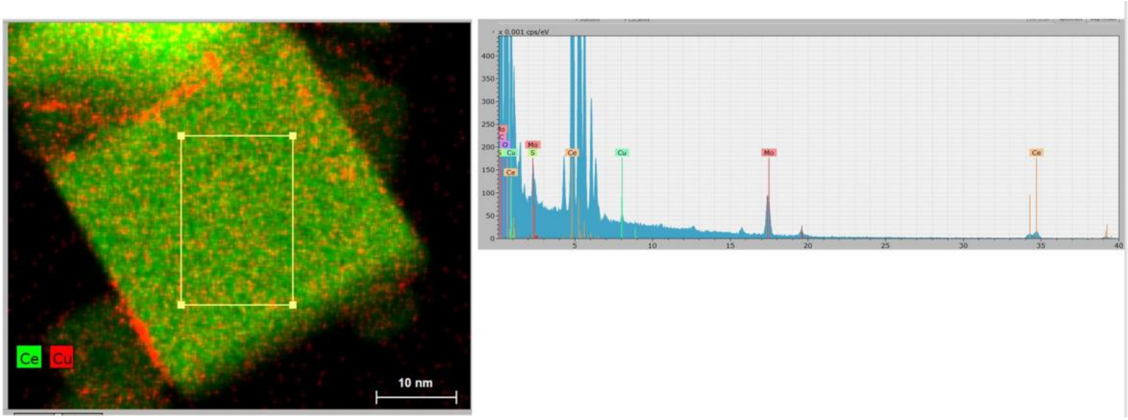

Figure S4. XEDS maps and spectrum from a relatively large area of the cube (approximately 140 nm^2, left) to confirm that, in regions on the (100) faces of the cubes where the copper signal appears very weak—practically at the noise level—there are indeed small amounts of copper present.

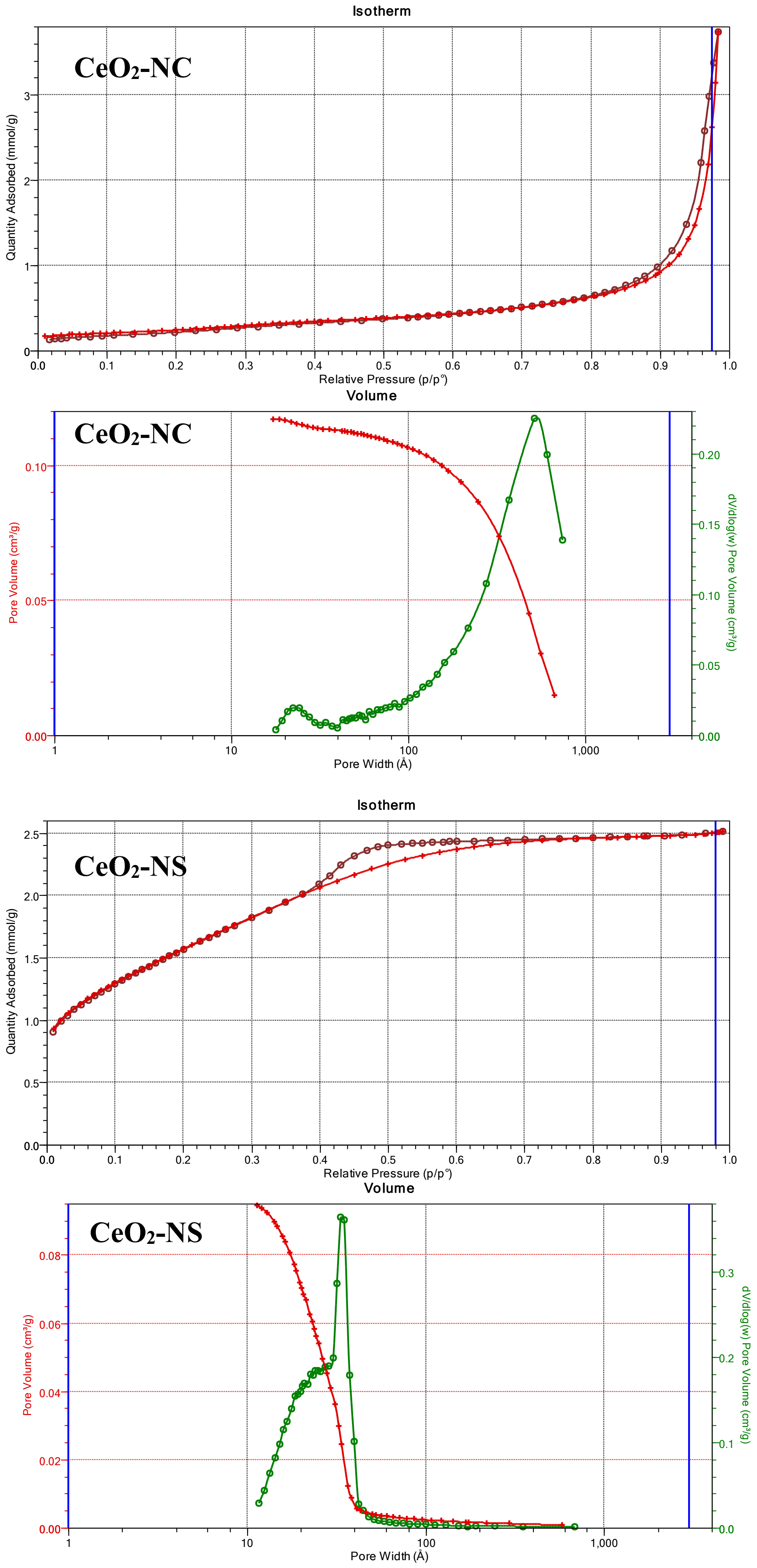

Figure S5. N_2 adsorption isotherms and Barrett-Joyner-Halenda (BJH) desorption pore size distribution curves for indicated supports.

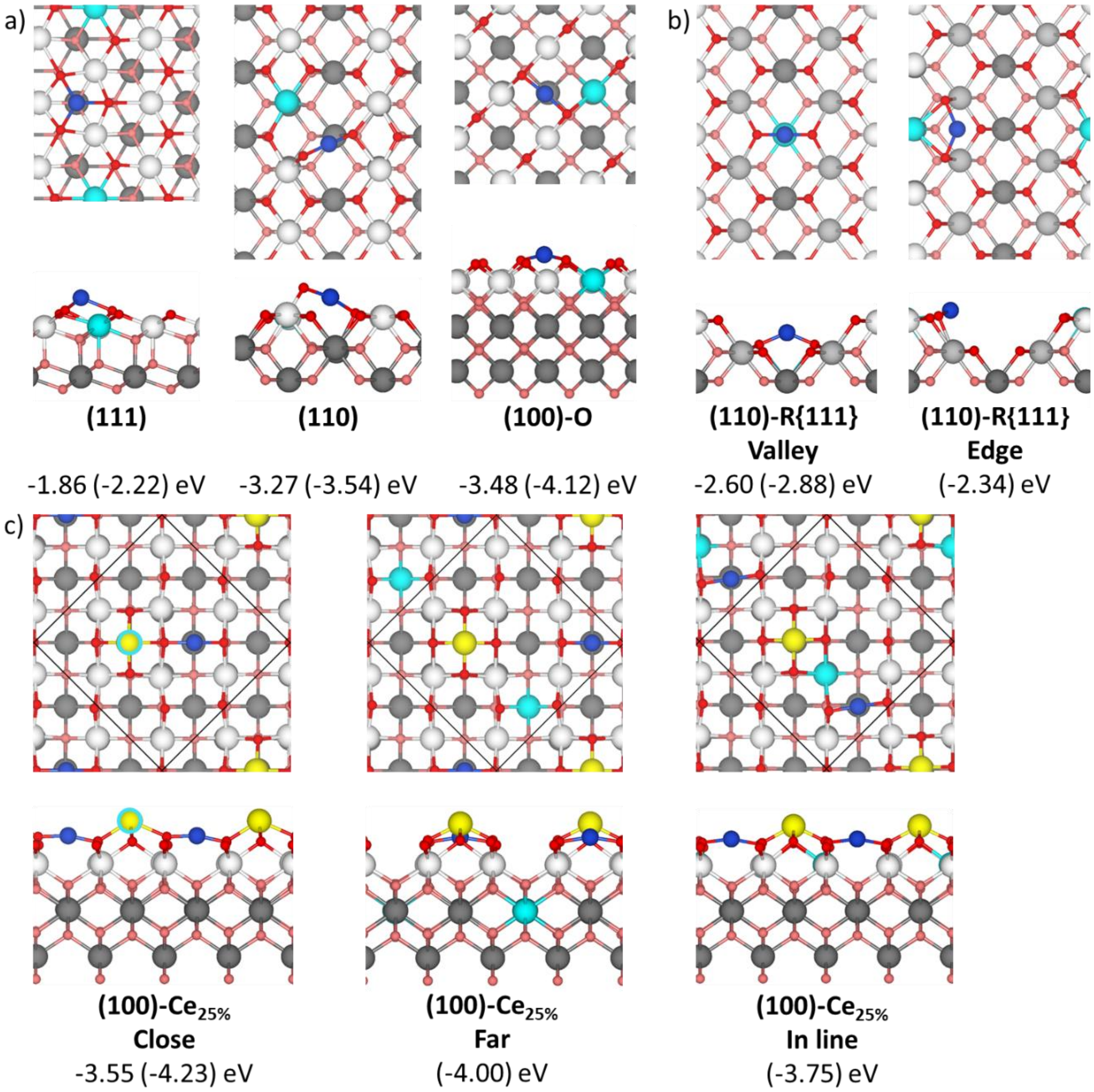

Figure S6. Reoptimized structures for a Cu atom adsorbed on the (111), (110) and (100)-O CeO_2 surfaces from our previous work [3] (a), alonside the optimized structures found in this work for a Cu atom adsorbed on the sawtooth-like (110)-R{111} CeO_2 reconstruction (b) and the partially Ce-terminated (100)-$Ce_{25\%}$ CeO_2 reconstruction (c). Energies calculated at the HSE level are indicated, with PBE energies provided in parenthesis. Surface Ce and O atoms are depicted in white and red, respectively, while deeper Ce and O atoms are depicted in grey and pink. Added Ce atoms forming CeO_4 pyramids are highlighted in yellow, Cu in dark blue, and Ce^{3+} in light blue.

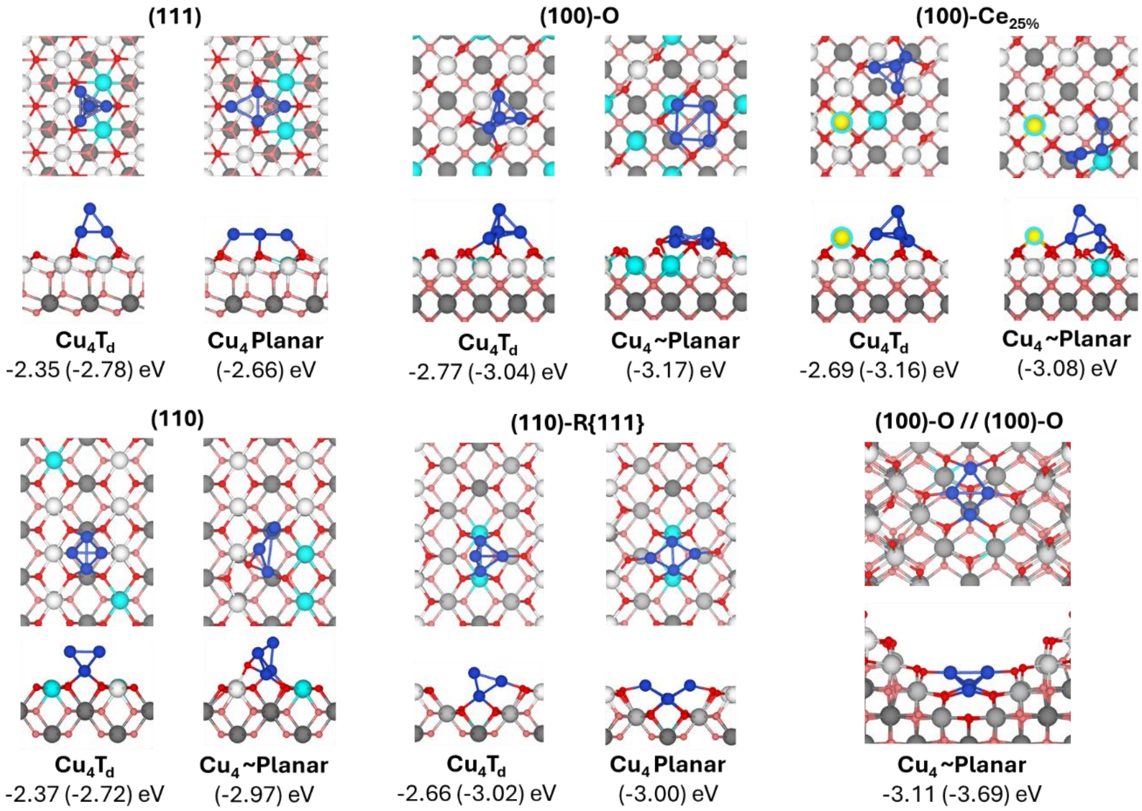

Figure S7. Most stable structures for the T_d and planar Cu_4 isomers adsorbed on the (111), (110) and (100)-O CeO_2 surfaces, the sawtooth-like (110)-R{111} and partially Ce-terminated (100)-$Ce_{25\%}$ CeO_2 reconstructions and the (100)-O // (100)-O model. Energies calculated at the HSE level are indicated, with PBE energies in parenthesis. Surface Ce and O atoms depicted in white and red, respectively, deeper Ce and O atoms in grey and pink. Added Ce atoms forming CeO_4 pyramids are highlighted in yellow, Cu in dark blue, and Ce^{3+} in light blue.

For the (111) and (110)-R{111} surfaces, the {111} matching between *111* facets of both Cu_4 T_d and planar isomers provide highly symmetric adsorbed structures, among which the Cu_4 T_d is more stable, in agreement with previous reports [4]. For the (100)-O surface, a lower energy distorted planar structure can be found, but it was found to break into a linear structure upon CO adsorption. Considering such result required a deeper analysis on the fluxionality of small copper clusters that was out of the scope of this work, and we used the Cu_4 T_d isomer for the CO study. Similarly, the most stable structure for the planar isomer on the (110) surface led to the planar upright structure with a lower energy w.r.t. the Cu_4 T_d structure reported (Figure S7), but it captures an O from the surface. While this result hints towards the oxidized state of a substantial proportion of copper in the experimental samples, the purpose of this DFT study is to model reduced Cu^+ species, and thus we used the Cu_4 T_d structure for the subsequent CO study. Attempts to reproduce the Cu_4 T_d structure from ref. [4] led to a similar less stable planar structure (third structure in Figure S9a). Also, note that the final $Cu_4T_d/CeO_2(110)$ reported and used in this work adopts an intermediate structure upon CO adsorption (Figure S8). For the (100)-$Ce_{25\%}$ reconstruction, the presence of the pyramid defect provides more different configurations that we explored for both isomers (Figure S9b). The Cu_4 T_d was more stable and we chose it for the CO study on this surface. Finally, we were not able to stabilize the Cu_4 T_d isomer on the (100)-O // (100)-O model, it always preferred an almost planar structure (Figure S9c).

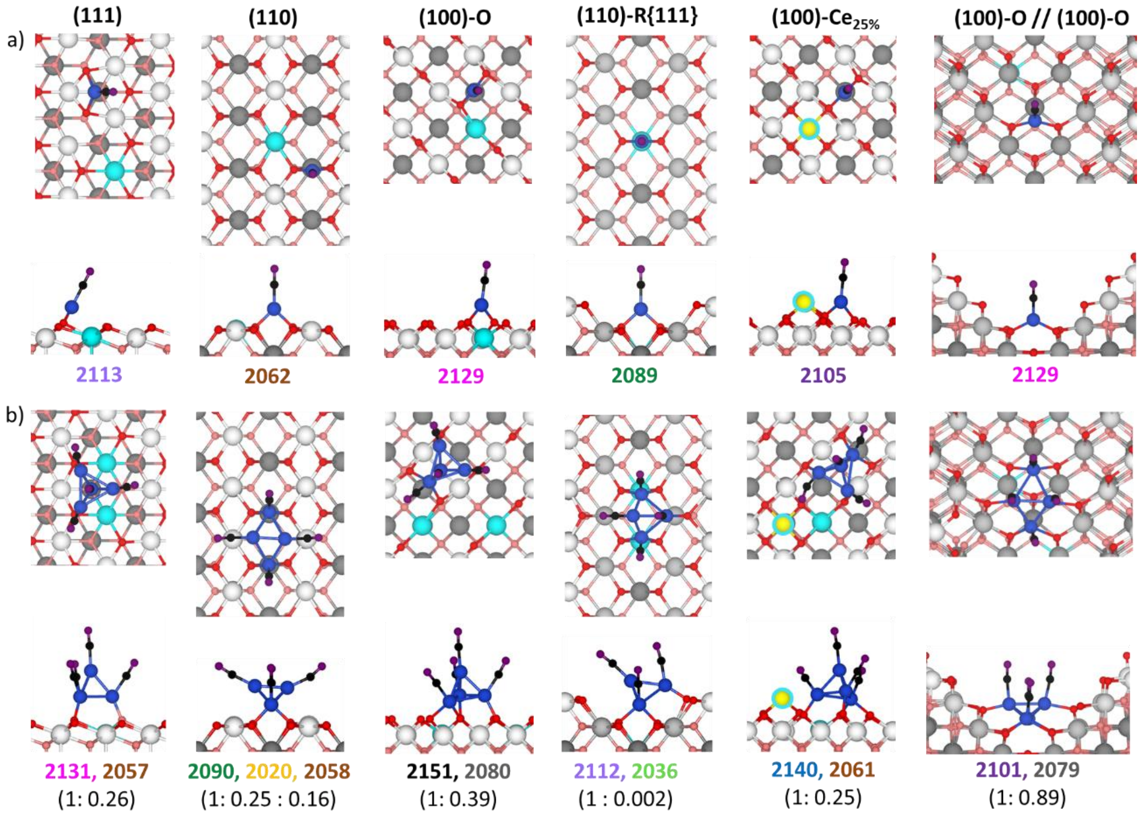

Figure S8. Top and side views for the optimized structures for CO adsorption on (a) a single Cu atom (Cu_1) and (b) a Cu_4 cluster on various ceria surfaces. CO vibrational frequencies calculated at the HSE06 level are shown in cm^{-1}, with colours corresponding to the experimental bands in Fig. 8. Relative intensities are also provided. Surface Ce and O atoms are shown in white and red, respectively; subsurface Ce and O atoms are rendered in grey and pink. Carbon and oxygen atoms from adsorbed CO are shown in black and purple, respectively. Ce atoms forming pyramidal reconstructions are highlighted in yellow, Cu atoms in dark blue, and Ce^{3+} sites in light blue.

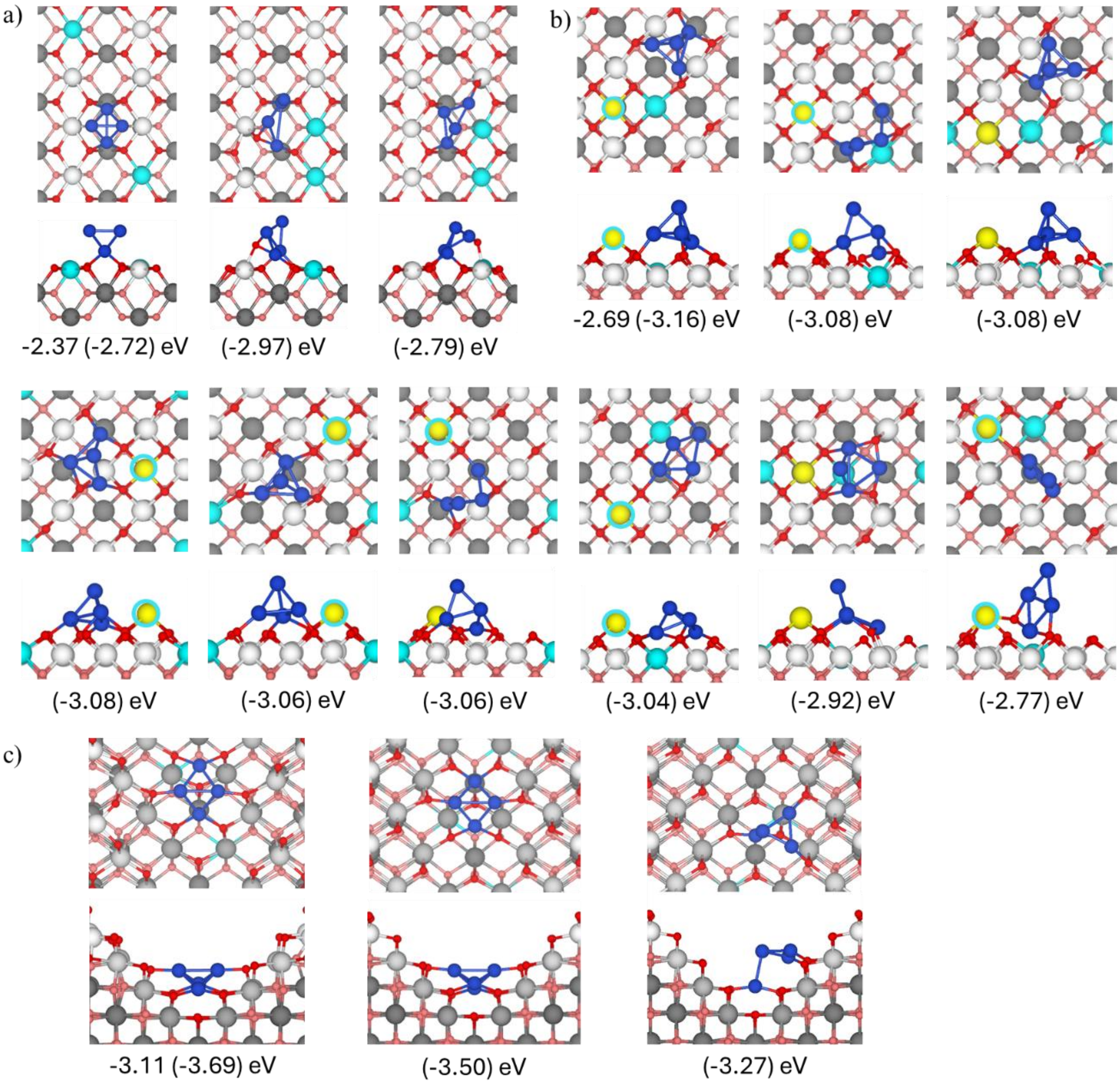

Figure S9. Top and side views for the optimized structures of Cu_4 clusters adsorbed on the (a) (110), (b) (100)-$Ce_{25\%}$ and (c) (100)-O // (100)-O models. Energies calculated at the HSE level are indicated, with PBE energies in parenthesis. Surface Ce and O atoms depicted in white and red, respectively, deeper Ce and O atoms in grey and pink. Added Ce atoms forming CeO_4 pyramids are highlighted in yellow, Cu in dark blue, and Ce^{3+} in light blue.

Supporting Information References

[1] Gamarra, D.; López Cámara, A.; Monte, M.; Rasmussen, S.B.; Chinchilla, L.E.; Hungría, A.B.; Munuera, G.; Gyorffy, N.; Schay, Z.; Cortés Corberán, V.; Conesa, J.C.; Martínez-Arias, A. Preferential oxidation of CO in excess H_2 over CuO/CeO_2 catalysts: Characterization and performance as a function of the exposed face present in the CeO_2 support. *Appl. Catal. B* **2013**, *130– 131*, 224– 238.

[2] Monte, M.; Gamarra, D.; López Cámara, A.; Rasmussen, S.B.; Gyorffy, N.; Schay, Z.; Martínez-Arias, A.; Conesa, J.C. Preferential oxidation of CO in excess H_2 over CuO/CeO_2 catalysts: Performance as a function of the copper coverage and exposed face present in the CeO_2 support. *Catal. Today* **2014**, *229*, 104–113.

[3] Pérez-Bailac, P.; Lustemberg, P.G.; Ganduglia-Pirovano, M.V. Facet-dependent stability of near-surface oxygen vacancies and excess charge localization at CeO_2 surfaces. *J. Phys.: Condens. Matter* **2021**, *33*, 504003

[4] Ren, Z.; Liu, N.; Chen, B.; Li, J.; Mei, D. Nucleation of Cu_n (n = 1–5) Clusters and Equilibrium Morphology of Cu Particles Supported on CeO2 Surface: A Density Functional Theory Study. *J. Phys. Chem. C* **2018**, *122*, 27402–27411.
